# Reconstructing and comparing signal transduction networks from single-cell protein quantification data

**DOI:** 10.1093/bioinformatics/btaf675

**Published:** 2025-12-23

**Authors:** Tim Stohn, Roderick A P M van Eijl, Klaas W Mulder, Lodewyk F A Wessels, Evert Bosdriesz

**Affiliations:** Department of Computer Science, Vrije Universiteit Amsterdam, Amsterdam, 1081 HV, The Netherlands; Division of Molecular Carcinogenesis, The Oncode Institute, The Netherlands Cancer Institute, Amsterdam, 1066 CX, The Netherlands; Department of Molecular Developmental Biology, Radboud Institute for Molecular Life Sciences, Radboud University, Nijmegen, 6500 GL, The Netherlands; Department of Molecular Developmental Biology, Radboud Institute for Molecular Life Sciences, Radboud University, Nijmegen, 6500 GL, The Netherlands; Division of Molecular Carcinogenesis, The Oncode Institute, The Netherlands Cancer Institute, Amsterdam, 1066 CX, The Netherlands; Faculty of EEMCS, Delft University of Technology, Delft, 2628 CD, The Netherlands; Department of Computer Science, Vrije Universiteit Amsterdam, Amsterdam, 1081 HV, The Netherlands

## Abstract

**Motivation:**

Signal transduction networks regulate many essential biological processes and are frequently aberrated in diseases such as cancer. A mechanistic understanding of such networks, and how they differ between cell populations, is essential to design effective treatment strategies. Typically, such networks are computationally reconstructed based on systematic perturbation experiments, followed by quantification of signaling protein activity. Recent technological advances now allow for the quantification of the activity of many (signaling) proteins simultaneously in single cells. This makes it feasible to reconstruct or quantify signaling networks without performing systematic perturbations.

**Results:**

Here, we introduce single-cell modular response analysis (scMRA) and single-cell comparative network reconstruction (scCNR) to derive signal transduction networks by exploiting the heterogeneity of single-cell (phospho-)protein measurements. The methods treat stochastic variation in total protein abundances as natural perturbation experiments, whose effects propagate through the network and hence facilitate the reconstruction and quantification of the underlying signaling network. scCNR reconstructs cell population-specific networks, where cells from different populations have the same underlying topology, but the interaction strengths can differ between populations. We extensively validated scMRA and scCNR on simulated data, and applied it to unpublished data of (phospho-)protein measurements of EGFR-inhibitor-treated keratinocytes to recover signaling differences downstream of EGFR. scCNR will help to unravel the mechanistic signaling differences between cell populations, and will subsequently guide the development of well-informed treatment strategies.

**Availability and implementation:**

The code used for scCNR in this study has been deposited on Zenodo https://doi.org/10.5281/zenodo.17600937 and is also available as a Python module at https://github.com/ibivu/scmra. Additionally, data and code to reproduce all figures is available at https://github.com/tstohn/scmra_analysis.

## 1 Introduction

Signal transduction networks play a vital role in cell physiology, where they regulate various biological processes like differentiation, proliferation, and apoptosis. Extracellular signals are commonly transmitted through intracellular networks of interacting proteins called kinases, which activate each other through post-translational modifications such as phosphorylation. Diseases such as cancer often are a consequence of aberrations in this signaling machinery (Hanahan and Weinberg 2011, Kolch *et al.* 2015), and many cancer drugs specifically target proteins within the signaling networks. However, adaptation and rewiring of the network (Ahronian *et al.* 2015) in response to treatment often limit the durability of a clinical response (Gerosa *et al.* 2020, Tognetti *et al.* 2021, Hoffman *et al.* 2023). Obtaining a mechanistic (Does protein A activate protein B?) and quantitative (How strong is the effect of protein A on protein B?) understanding of those networks, and how they differ between cell populations (such as resistant or mutant cells), is a key challenge in cellular biology and has important implications for the design of treatment strategies (Klinger *et al.* 2013, Bosdriesz *et al.* 2022).

Various methods have been developed to solve this problem. Models based on ordinary differential equations are detailed and quantitative (Fey *et al.* 2015, Raue *et al.* 2015), and have been extended to large systems (Schmiester *et al.* 2020). Nevertheless, these methods rely on numerous measurements to fit the models. Boolean logical network models offer a simpler, more abstract way to describe networks (Saez-Rodriguez *et al.* 2009, Hill *et al.* 2012, Oates *et al.* 2012, Grieco *et al.* 2013) and have been extended to explain quantitative information (Landtsheer *et al.* 2017) and heterogeneous population dynamics (Calzone *et al.* 2022). Modular response analysis (MRA) finds the middle ground by modeling networks quantitatively without the complexity of fully dynamical models (Bruggeman *et al.* 2002, Kholodenko *et al.* 2002). MRA determines the interaction strengths between proteins based on systematic perturbation experiments, in which the states of all nodes in the network are recorded before and after the perturbations. MRA is able to detect cross-talks and feedback loops, and has been successfully employed to quantify novel network topologies (Klinger *et al.* 2013, Dorel *et al.* 2018). Various alternative formulations have been developed, such as optimization-based approaches (Bosdriesz *et al.* 2018), maximum-likelihood approaches (Klinger *et al.* 2013) or Bayesian methods (Santra *et al.* 2013, Halasz *et al.* 2016, Rukhlenko *et al.* 2022).

While most methods model signaling networks in a specific context, differences between networks derived from different contexts are often most informative. For example, comparing networks derived from a cell line before and after acquiring resistance to a targeted inhibitor can aid in elucidating the signaling changes that drive the resistance mechanism (Bosdriesz *et al.* 2018). Similarly, models of cell lines with and without a particular oncogenic mutation can help in prioritizing drug combinations that are specific to a particular genetic background (Bosdriesz *et al.* 2022). Finally, modeling the dependence of signaling networks on cell states can help predict interventions that control cell fate decisions (Rukhlenko *et al.* 2022). To compare networks across contexts, we previously developed comparative network reconstruction (CNR) (Bosdriesz *et al.* 2018), which aims to identify quantitative differences between the signaling networks derived from cell populations in different contexts.

Most methods that model signal transduction networks were developed for bulk data. However, even in isogenic populations in a homogeneous environment, cells in different cell states are known to respond differently to the same instructions (Kim *et al.* 2018, Kramer *et al.* 2022, Wang *et al.* 2022). This has important implications for drug resistance and cancer treatment design (Korkut *et al.* 2015, Aissa *et al.* 2021) and efforts have been undertaken to link signaling networks to their effect on cell state transitions and cell fate (Rukhlenko *et al.* 2022). Obtaining such insights has long been elusive because we lacked the appropriate data to study them. However, recent development of technologies for high-dimensional single-cell quantification of (phospho-)proteins and post-translational modifications, based on mass cytometry (Tracey *et al.* 2021), DNA-barcoded antibodies (Stoeckius *et al.* 2017, Eijl *et al.* 2018, Sheng *et al.* 2022, Ariss *et al.* 2024, Opzoomer *et al.* 2024) and spatial methods such as iterative indirect immunofluorescence imaging (Gut *et al.* 2018) now allow, for the first time, to study and model the mechanisms underlying the heterogeneity of signal transduction between cell populations in a data-driven manner.

Methods for reconstructing signaling networks have increasingly incorporated such single-cell data. For instance, several recent studies derive population or cell line-specific networks from single-cell data (Brandt *et al.* 2019, Tognetti *et al.* 2021, Rukhlenko *et al.* 2022). However, these methods all use the single-cell nature of the data only to cluster cells in distinct groups, and then aggregate the cells within one population. As such, they do not exploit the within-population variation in signaling activity and they require many perturbations for network inference or quantification. The concept of leveraging single-cell protein variation to extract biological insights has been previously discussed (Slavov, 2022) and the DREMI algorithm uses cell-to-cell variation in protein activity to gain insight into the strength of protein interactions (Krishnaswamy *et al.* 2014). However, this method is limited to analyzing protein pairs and does not extend to network reconstruction. Knowledge-primed neural networks, which integrate prior biological knowledge, have been employed to infer pathways from single-cell RNA-seq data (Fortelny and Bock 2020), and Sachs *et al.* (2005) used a Bayesian framework to infer causal interaction from high-dimensional flow cytometry data, but both these methods are restricted to acyclic networks, thus overlooking commonly occurring feedback loops.

To fully exploit heterogeneity between individual cells, we developed single-cell MRA (scMRA) and single-cell CNR (scCNR). These methods exploit the fact that protein levels vary across cells due to stochastic differences, heterogeneity in gene expression, post-transcriptional processes, or other regulatory mechanisms (Sigal *et al.* 2006, Budnik *et al.* 2018, Lanz *et al.* 2022, Khan *et al.* 2025). This variation in total protein will typically translate into a variation in the corresponding phospho-protein, resulting in cell-to-cell variability in the abundance of phosphoproteins. For instance, in the simple case of a protein phosphorylation site with constant phosphorylation and de-phosphorylation rates, the (steady-state) phospho-protein abundance is a constant fraction of the total protein abundance, and an increase in total protein abundance will result in a proportional increase of the abundance of the phosphorylated protein (c.f. Supporting Information 3, available as supplementary data at *Bioinformatics* online). Since the timescale at which proteins vary (hours to days; Sigal *et al.* 2006) is much longer than the timescale at which protein phosphorylation occurs (seconds to minutes; Ahmed *et al.* 2014, Ryu *et al.* 2018, Kim *et al.* 2023), the corresponding phospho-protein levels are in a quasi-steady state. Together, this implies that the cell-to-cell variation in total protein abundances can be modeled as natural perturbations to their corresponding phospho-proteins, where the perturbations are relative to the population average. These “perturbations” in phosphoprotein levels will in turn affect the phosphorylation of downstream proteins, and as such propagate throughout the network.

Much like traditional MRA and other “perturbation biology” related methods (Kholodenko *et al.* 2002, Korkut *et al.* 2015, Bosdriesz *et al.* 2018, Dorel *et al.* 2018) developed for bulk data, scMRA is a formalism that allows to disentangle the direct perturbation effects and indirect network effects and as such allow for inference of causal interactions. However, in contrast to the bulk method, it does not require extensive perturbation effects. Note that scMRA does not reconstruct a unique network for every single cell, but rather reconstructs a single network for the whole population of cells. By considering each single cell as a data-point, scMRA vastly increases the number of observations from which the reconstructions are derived. Typically, differences in signaling between cell populations is of most interest, e.g. due to cell state effects such as the cell cycle and differentiation, or due to treatment effects or disease progression or the emergence of resistant subpopulations. Therefore, we extended scMRA to scCNR, in order to identify which interactions differ quantitatively between cell populations. Similar to CNR (Bosdriesz *et al.* 2018), scCNR reconstructs a single shared network topology with cell population-specific interaction strengths.

We extensively validated scMRA and scCNR on simulated single-cell data of the epidermal growth factor receptor (EGFR) signaling pathway and showed that both methods perform well using as few as hundred cells as input, and in the presence of considerable noise. Additionally, we quantified 70 (phospho)proteins of key signaling nodes in EGFR-inhibitor-treated keratinocytes using single-cell ID-seq (Eijl *et al.* 2018). We show that scCNR recovers meaningful treatment-induced differences of protein interactions downstream of EGFR and related to proliferation.

## 2 Materials and methods

### 2.1 Formulation of scMRA and scCNR

**Figure 1.**
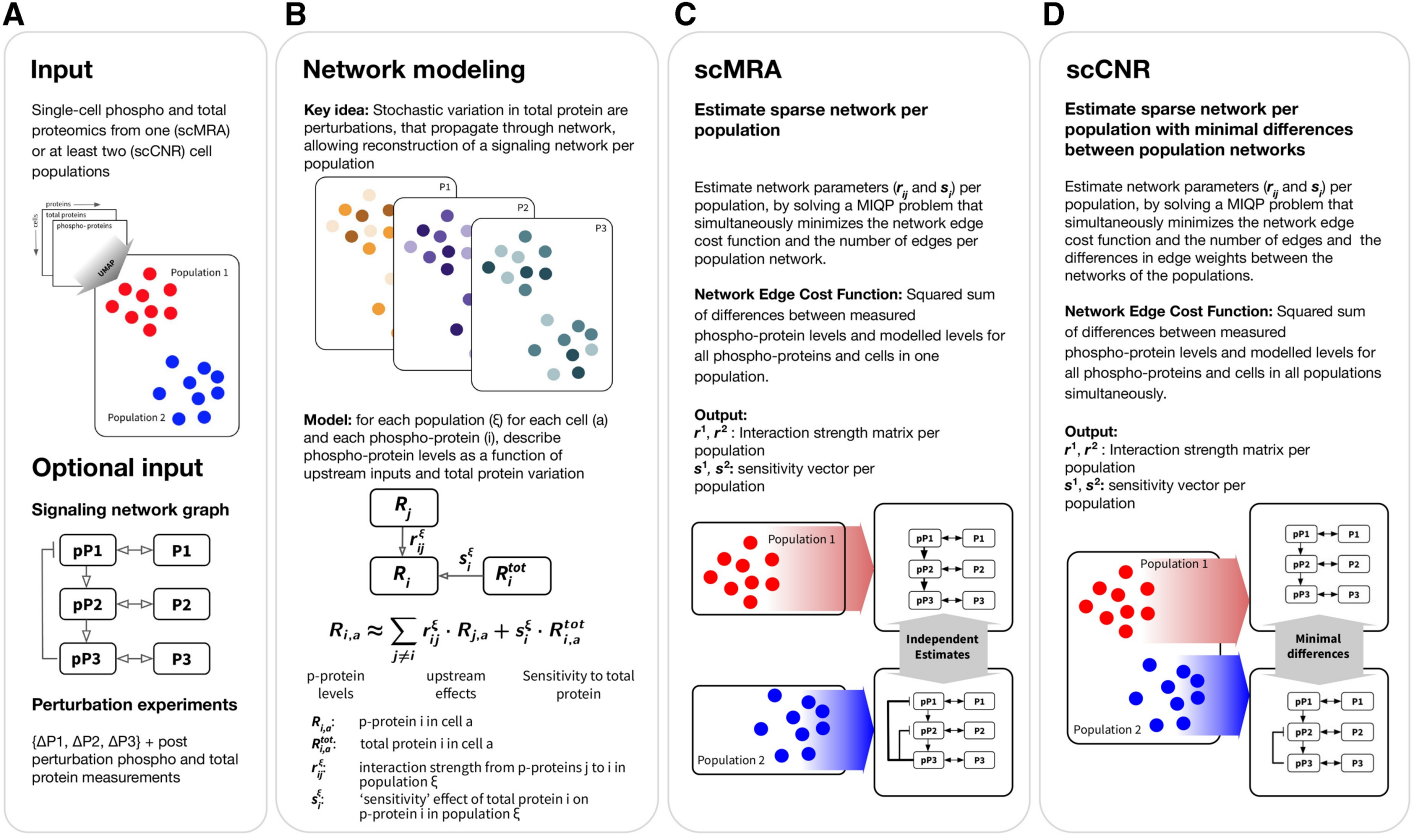
Schematic overview of scMRA and scCNR. (A) The methods take phospho- and total protein abundances from single cells as input, with additional cell population annotations for scCNR. Optionally, they can be enriched with perturbation data and a prior network topology. (B) The methods exploit natural heterogeneity of phospho- and total protein abundances between cells to infer the network topology and quantify the interaction strengths between network nodes. They fit the data to describe single-cell deviations of phosphoprotein from its population mean (*R*) for a single population (scMRA) or several populations in parallel (scCNR) to derive (cell population-specific) interaction strengths (*r*). (C, D) Both algorithms penalize the number of edges in the network. (D) scCNR further penalizes the number of population-specific interaction strengths.

Here, we briefly describe the formalism underlying scMRA and scCNR. More details and a full derivation of the equations, including the underlying assumptions, can be found in the Supplementary Information 2 and 3, available as supplementary data at *Bioinformatics* online. scMRA and scCNR exploit stochastic variation in protein abundances to identify and quantify the interaction strengths between different nodes in a signal transduction network. As input, they require single-cell measurements of the abundance and activity of all nodes in the network, and if applicable, the applied perturbations. Through a first-order Taylor expansion around the bulk-average steady-state, these are related through the following set of linear equations (one for each protein *i* in each cell *a*):


(1) $$R_{i,a}\approx \sum_{j\neq i}r_{ij}^{\xi }\cdot R_{j,a}+s_{i}^{\xi }\cdot R_{i,a}^{\mathrm{tot}}+\sum_{m}p_{im}^{\xi }\;\forall a,i.$$

where $x_{i,a}$ is the measured abundance of active protein *i* in cell *a* and $R_{i,a}\equiv (x_{i,a}-\langle x_{a}^{\xi }\rangle )/\langle x_{a}^{\xi }\rangle$ is the deviation of $x_{i,a}$ from the average of its population ξ, $\langle x_{a}^{\xi }\rangle$. This measurable deviation of the activity of protein *i* is explained by deviations in the activity of upstream proteins (indexed by *j*) as well as the sensitivity of the activity of protein *i* to changes in its total protein abundance (the natural perturbation effect) (Fig. 1B), and potentially additional perturbations *m* to protein *i* (as in traditional MRA). $R_{i,a}^{\text{tot}}\equiv (x_{i,a}^{\text{tot}}-\langle x_{a}^{\text{tot},\xi }\rangle )/\langle x_{a}^{\text{tot},\xi }\rangle$ is the deviation of the measured total protein abundance $x^{\text{tot}}$ from the average of the population ξ, $r_{ij}^{\xi }\equiv \frac{\langle x_{j}^{\xi }\rangle }{\langle x_{i}^{\xi }\rangle }\frac{\partial x_{i}^{\xi }}{\partial x_{j}^{\xi }}$ is the population-specific interaction strength between protein *j* and protein *i*, $s_{i}^{\xi }\equiv \frac{\langle x_{i}^{\text{tot},\xi }\rangle }{\langle x_{i}^{\xi }\rangle }\frac{\partial x_{i}^{\xi }}{\partial x_{i}^{\text{tot},\xi }}$ the population-specific sensitivity of protein *i* activity to a change in total protein, and $p_{im}^{\xi }\equiv \frac{p_{m}}{\langle x_{i}^{\xi }\rangle }\frac{\partial x_{i}^{\xi }}{\partial p_{m}}\frac{\Delta p_{m}}{p_{m}}$ is the direct effect that perturbation *m* has on the activity of protein *i* in the population ξ.

scMRA and scCNR both solve a Mixed Integer Quadratic Programming (MIQP) optimization problem that aims to find the values of $r_{ij}^{\xi }$, $s_{i}^{\xi }$ and $p_{im}^{\xi }$ that minimize the squared error, $\epsilon_{i,a}$, representing the deviations between measured active protein abundance per cell ($R_{i,a}$) and the prediction of the model as described by the right-hand side of equation (1). scMRA and scCNR both add an $L_{0}$-regularization penalty on the total the number of interactions in the network. scCNR has an additional $L_{0}$-regularization penalty on the number of population-specific interaction strengths, sensitivities to change in total protein abundance, and perturbation effects. The full MIQP formulation for scCNR then reads as follows:


$$\begin{array}{c} \text{minimize:}\sum_{a,i,j,m}\left[\left(1-\eta )\cdot \epsilon_{i,a}^{2}+\eta \cdot I_{ij}^{\text{edge}}+\theta (I_{ij}^{r}+I_{i}^{s}+I_{im}^{p}\right)\right]\\ \text{subject}\;\text{to:}\sum_{j=1}^{N_{n}}r_{ij}^{\xi }\cdot R_{j,a}+s_{i}^{\xi }\cdot R_{i,a}^{\text{tot}}+\sum_{m=1}^{N_{p}}p_{im}^{\xi }=\epsilon_{i,a}\;\forall a,i\\ I_{ij}^{\text{edge}}=0\Rightarrow r_{ij}^{\xi }=0\;\forall i,j,\xi \\ I_{ij}^{r}=0\Rightarrow r_{ij}^{\xi }=\langle r_{ij}\rangle \;\forall i,j,\xi \\ I_{i}^{s}=0\Rightarrow s_{i}^{\xi }=\langle s_{i}\rangle \;\forall i,\xi \\ I_{im}^{p}=0\Rightarrow p_{im}^{\xi }=\langle p_{im}\rangle \;\forall i,m,\xi \\ I_{ij}^{\text{edge}},I_{ij}^{r},I_{i}^{s},I_{im}^{p}\in \{0,1\} \end{array}$$

where $r_{ii}\equiv -1$, $N_{n}$ is the number of nodes in the network and $N_{p}$ the number of perturbations. $I_{ij}^{\text{edge}}$ is a binary indicator for the presence or absence of an interaction between nodes *i* and *j*. This indicator is the same across all cell populations since we recover one shared network topology, and its presence in the objective function penalizes the number of edges in the network. Prior information about the presence of an edge from protein *j* to *i* can be incorporated by a constraint $I_{ij}^{\text{edge}}=1$. In scCNR, the strength of the interaction between two nodes is allowed to differ between populations, but scCNR penalizes such population-specific interaction strengths (i.e. edges that are different between the populations), population-specific sensitivities to total protein change and perturbation effects with the binary indicators $I_{ij}^{r}$, $I_{i}^{s}$ and $I_{im}^{p}$, respectively. The hyperparameters η and θ determine the relative weight of the regularization penalties. For the inclusion of perturbation effects see Supplementary Information 4.2, available as supplementary data at *Bioinformatics* online. The optimization problem was solved using IBM ILOG CPLEX solver (version 20.1.0). CPLEX is available free of charge for academic purposes and guarantees an optimal solution within small numerical tolerances for our problem.

### 2.2 Simulating single-cell protein data

Single-cell protein abundance data were simulated using an existing ODE-based dynamic model of the EGFR signaling pathway (Orton *et al.* 2009), which we re-implemented in Wolfram Mathematica (Wolfram Research, Inc. 2019). Single-cell total protein variation was simulated by multiplying the abundance of each protein used in the original model with a random variable sampled from a log-normal distribution (with μ=0 and σ=0.1), and than calculating the resulting quasi-steady state of the active proteins. Steady states were calculated by explicitly calculating the active protein concentrations for which the left-hand side of all ODEs vanishes. To this end, we first approximated the steady state by integrating the ODEs using the NDSolve function, which was used as input to subsequently calculate the exact numerical solution using the FindRoot function. (Drug) perturbations were simulated as a 25% decrease in the catalytic activity of the corresponding protein-activating reactions. BRAF and RAS mutations were modeled as a 100-fold reduction in the deactivation rate of active BRAF and RAS, respectively. Noise was added to the input data by multiplying the data with a random number drawn from a normal distribution with unit mean and an SD equal to the noise. The ground truth interaction strengths between proteins of the Orton model were numerically calculated as the partial derivatives of active protein with respect to its upstream active protein. The simulations, along with the numerically calculated true interaction strengths, are available in the “simulations” directory of the GitHub repository associated with the paper’s analyses: https://github.com/tstohn/scmra_analysis.

### 2.3 scID-seq analysis

For full details, see Supplementary Information 1.2–1.6, available as supplementary data at *Bioinformatics* online. Briefly, primary pooled human epidermal stem cells were treated with the EGFR inhibitor AG1478 or vehicle (DMSO) for 48 h. Subsequently, the abundance of 70 phosphorylated and total proteins in 282 cells was quantified using single-cell ID-seq (Eijl *et al.* 2018). We ran scCNR to detect signaling differences between the untreated and EGFR-inhibitor-treated cell population and used a prior literature-derived network topology.

## 3 Results

### 3.1 scMRA and scCNR reconstruct networks from total protein variation in single-cell protein data

To reconstruct signal transduction networks from the quantification of phospho- and total proteins in single cells, we developed scMRA. The underlying formalism is based on three biologically motivated assumptions (c.f. Supplementary Information 3, available as supplementary data at *Bioinformatics* online): (i) that stochastic differences in the abundance of total proteins linearly affect the abundance of the corresponding phosphoproteins, (ii) that effects on other phosphoproteins are mediated through changes in the abundance of its phosphorylation form, and (iii) that the timescale at which total proteins abundances fluctuate is much slower than the timescale at which phosphorylation takes place (Sigal *et al.* 2006, Ahmed *et al.* 2014, Ryu *et al.* 2018, Kim *et al.* 2023). The model assumes local linearity around the steady state for (i) interactions between total and phosphorylated measurements of a protein, and (ii) interactions between phosphoproteins. Taken together, this implies that stochastic variability of total protein levels between single cells can be modeled as natural perturbations to the corresponding phosphoprotein, which will subsequently propagate through the network leading to distinct steady states in each individual cell (Fig. 1A and B). scMRA employs the measurements of each cell as independent measurements to perform a reconstruction of the underlying signaling network for the whole population, where nodes of the network represent phosphoproteins and edges represent the interaction strengths between phosphoproteins.

scMRA reconstructs a unique set of interaction strengths for each cell population, such as the red and blue cells representing populations that can be annotated from prior-knowledge about the input cells or can be derived by clustering of the data (Fig. 1C). Cell populations may represent, for instance different cell states, cells with and without an oncogenic mutation, cells before and after acquiring resistance to a drug, or cells that are cultured for a long time in the presence or absence of an inhibitor. To focus on the variation between cell populations, we developed scCNR to identify population-specific interaction strengths, just as CNR models condition-dependent interaction strengths in the bulk setting (Bosdriesz *et al.* 2018). This allows for the identification of the most relevant differences between the cell populations.

Input to the methods are deviations of abundances of total ($R^{\text{tot}}$) and phosphoprotein (*R*) levels of each cell from the cell population mean, for each node in the network (Fig. 1B). The output is the network topology described by interaction strengths between phosphoproteins (*r*), and sensitivities of phosphoproteins to deviations in the level of its total protein (*s*) (Fig. 1B). scCNR fits population-specific models that share the same network topology, i.e. share the same nodes and the same interactions between nodes. However, the interaction *strengths* are allowed to differ between the population-specific networks. scCNR is formulated as an MIQP problem and fits a model that (i) for each node in each cell aims to explain the deviations of phosphoprotein abundance from the population mean; (ii) penalizes the number of interactions (model complexity); and (iii) penalizes the number of population-specific interaction strengths (Fig. 1D).

Often, not all total protein abundance measurements are available for all nodes in the network. However, additional perturbations, e.g. by small molecular inhibitors, can be included in the experimental design and model to facilitate network reconstruction. Furthermore, the formulation of the algorithm allows for easy integration of prior network information by adding these as binary indicator constraints to the optimization problem, for instance; in cases where the topology is partly established and the main interest lies in quantifying the interaction strengths (Fig. 1A).

### 3.2 scMRA faithfully reconstructs the topology of signal transduction networks

**Figure 2.**
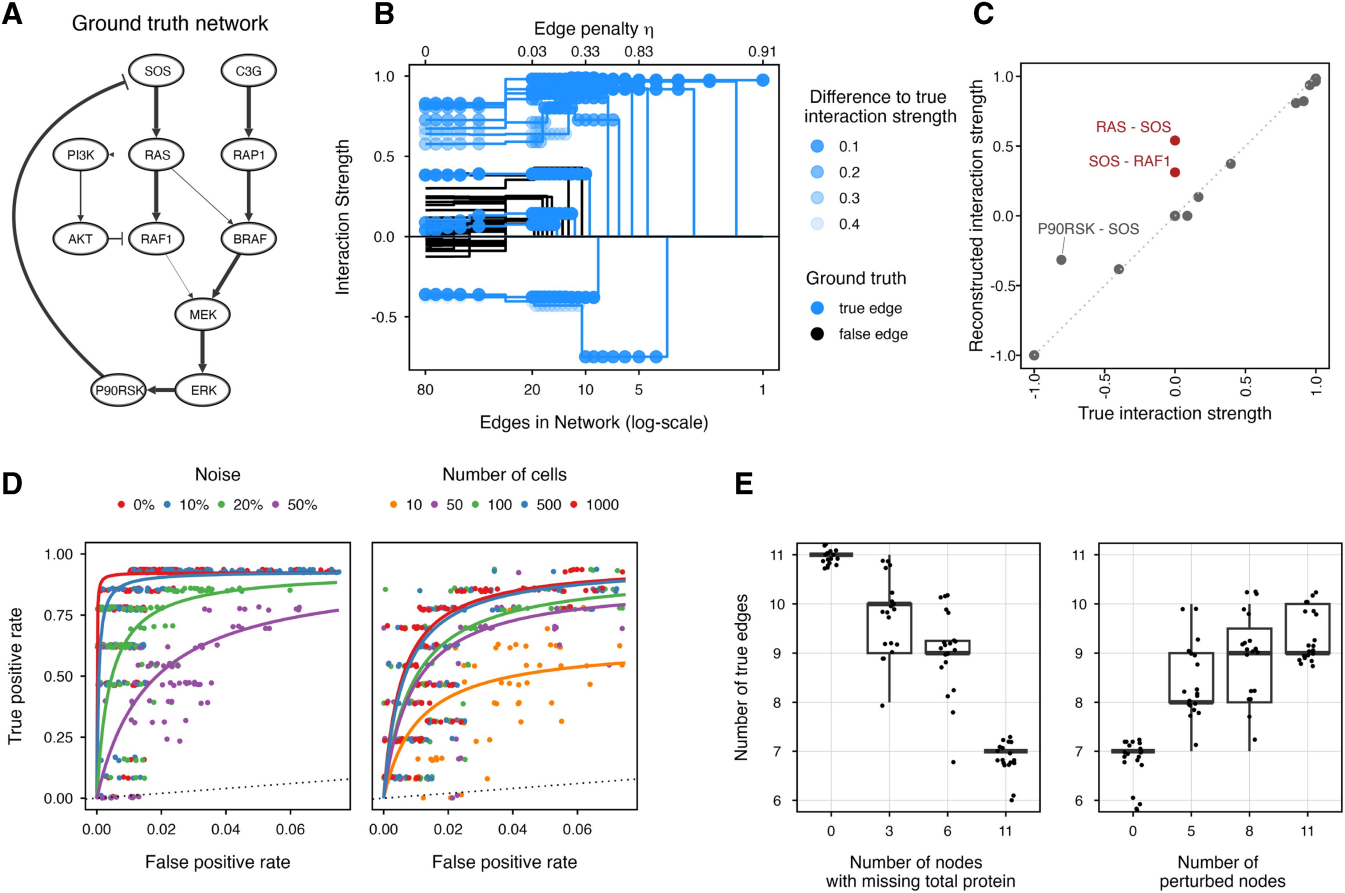
Evaluation of scMRA. (A) The EGFR signaling pathway according to the Orton model. Arrow width is equivalent to the interaction strength derived from the Orton model. (B) Interaction strengths reconstructed using scMRA for reconstructions of decreasing network complexity (increasing edge penalty η). True positives are indicated in blue and false positives in black. Transparency indicates the difference to the true interaction strength. (C) Correlation between true and reconstructed interaction strengths for a network reconstruction with 1000 cells and 20% noise. (D) Receiver-operating characteristic curves for the effect of noise and number of cells on the reconstruction of the network topology. (E) Number of true edges recovered in the scMRA-reconstructed networks as a function of the number of nodes with missing total protein measurements (left panel) and as a function of the number of perturbed nodes (right panel). These graphs are based on simulations with randomly removed total protein measurements (left panel) and simulations with completely absent total protein measurements but additional perturbations for randomly selected nodes (right panel).

To evaluate how well scMRA recovers network topology and node interaction strengths, we first set out to test it on simulated data for which the ground truth is known. To this end, we simulated single-cell data for the EGFR signaling pathway using a previously published dynamic model described by ordinary differential equations (Orton model), and reconstructed the network (Orton *et al.* 2009) (Fig. 2A). Note that since scMRA and scCNR aim to explain the steady-state deviation of phosphoprotein abundance from the cell population mean only, the scMRA and scCNR network reconstructions will be much simpler than the original model. Nevertheless, the true interactions between active proteins are unambiguously defined (Fig. 2A). We simulated single cells by sampling total protein abundances from a log-normal distribution, and calculated the corresponding phosphoprotein abundances at the steady state.

As a first test, we simulated 1000 cells, and added 20% noise to the data. We repeatedly reconstructed the signaling network using scMRA and progressively increased the complexity penalty η, resulting in increasingly sparse networks. Note that we did not include any prior network information. Figure 2B shows the reconstructed interaction strengths of the network, with true positive edges indicated in blue and false positives in black. Importantly, when increasing the η-penalty, false positive edges are the first to be eliminated, while the majority of true positive edges are retained. Furthermore, in reconstructions with low η-penalties and thus many edges, the false positive edges have typically weaker interaction strengths than the true positive edges. However, in reconstructions with a low η-penalty the strengths of the true positive edges are typically slightly different from their true values, as indicated by the transparency of the points in Fig. 2B. As the penalty increases and false positive edges vanish, the reconstructed true edges in the network converge toward their actual interaction strength. This is further exemplified by the strong correlation between reconstructed and true interaction strengths (Fig. 2C).

### 3.3 scMRA works well in the presence of noise and with few input cells

As single-cell data are inherently noisy, we investigated how this impacts the performance of scMRA by adding varying amounts of noise on the input data. Similar to the analysis described above, we repeatedly reconstructed the Orton network while decreasing the network connectivity (by increasing the η parameter). Comparing the recovered edges to the ground truth network, we generated receiver-operating characteristic (ROC) curves. With 20% noise a true positive rate exceeding 75% can be achieved while keeping the false positive rate below 2.5% (Fig. 2D, left panel, note that the *x*-axis only extends to 0.075). Even with as much as 50% noise added to the data, a 70% true positive rate is attained at a false positive rate below 6%. To put the numbers into perspective: a reconstructed network with 13 edges from a simulation with 1000 cells and 20% noise yields a network with two false positive edges (Fig. S1A, available as supplementary data at *Bioinformatics* online). In addition, the reconstructed interaction strengths are very similar to their true values, and only two edges with weak interaction strengths, namely RAS-PI3K and RAF1-MEK, are not recovered (Fig. 2C).

Profiling large numbers of cells is not always feasible, as for instance even in large datasets specific cell populations of interest might be underrepresented. Hence, we tested the influence of the number of cells measured on the performance of scMRA by simulating populations of various sizes, each with 20% noise added. The resulting ROC curves are shown in Fig. 2D, right panel. For 500 cells or more, a true positive rate above 75% at a 2.5% false positive rate is attained. With as few as 50 cells a true positive rate of 75% with a 5% false positive rate can be attained. In cases where noise levels are too high and/or cell numbers too limited to achieve satisfactory results, scMRA network reconstruction can be improved by adding prior network information (Fig. S2, available as supplementary data at *Bioinformatics* online). Taken together, this illustrates that scMRA can accurately reconstruct the network from few cells in the presence of considerable noise.

### 3.4 Perturbation experiments can compensate for missing total protein measurements

Ideally, scMRA uses measurements of phospho- and total protein for all nodes in the network, but this might not always be feasible due to experimental constraints. To assess the sensitivity of scMRA to missing total protein measurements, we removed total protein information of randomly selected proteins from the simulated data. We ran simulations of 1000 cells with 20% noise and counted true and false edges in the scMRA-reconstructed networks. To allow a fair comparison between reconstructed networks, we compared networks with 13 edges (the number of edges in the true network).

As expected, increasing the number of proteins for which their total abundance is missing decreases the number of true edges in the recovered networks (Fig. 2E, left panel) from 11 edges on average when all total proteins are measured to only seven edges when all total proteins are missing. To compensate for missing total protein measurements, scMRA can input perturbation data as input to improve network identification. To examine to which extent this could help to mitigate the negative effect of missing total protein, we simulated inhibitor treatment perturbations to a node as a 25% reduction in the catalytic activity of reactions activating that node. We randomly selected nodes to be perturbed, performed the perturbations, and then reconstructed the network. When including the perturbations in the reconstruction, we fixed the total number of cells to 1000. Adding perturbation data can increase the average number of true edges from seven to nine edges in complete absence of total protein (Fig. 2E, right panel). This confirms that additional perturbations can partially compensate for missing total protein measurements and could be an option to include in the experimental design when total protein measurements might not be fully available.

### 3.5 scCNR identifies signaling differences between simulated wild-type and mutant cell populations

In many biological contexts, the differences between cell populations are of primary interest—for example, understanding how signaling interactions differ quantitatively between populations or why one population reacts differently to a treatment than another. To enable the investigation of differences in signaling between such populations, we developed scCNR. scCNR is based on CNR (Bosdriesz *et al.* 2018) that aims to identify the most important quantitative differences between cell populations. Cell populations can be assigned by clustering the data or manual annotation prior to network reconstruction. To this end, in scCNR, we reconstruct a single network topology for all populations simultaneously, while allowing distinct interaction strengths for each population. In other words, scCNR outputs one shared network topology with a set of population-specific interaction strengths. In addition to the complexity penalty on the number of edges (η), scCNR also penalizes the number of population-specific interaction strengths (θ). This greatly reduces the total number of model parameters, thereby reducing overfitting and focusing on the most important differences. An additional advantage of this approach is that the reconstruction of each population is based on the full dataset, hence allowing information to be “borrowed” between cell populations.

We evaluated scCNR on simulated single-cell populations of mutant RAS and BRAF cells, which we compared to the simulated wild-type population. These mutations cause constitutive activation of RAS and BRAF, respectively, which we modeled similarly to Orton *et al.* (2009) by a 100-fold reduction of the deactivation rate of the mutant protein. Like in the evaluation of scMRA, we used as input for scCNR only the (phospho)protein profile without any prior network topology. We simulated 250 cells per population and added 20% noise. We ran up to 30 preliminary simulations with a range of η to obtain networks with the number of edges close to 13, in order to compare networks of similar connectivity. Next, we estimated an appropriate value for θ, which reduces the residuals of the objective function while keeping the model complexity low (Fig. S3A and B, available as supplementary data at *Bioinformatics* online). We estimated θ from one example simulation and due to the stochastic nature of the simulations, the exact number of population-specific edges varies slightly across simulations. This resulted in approximately four edges that differed between the mutant and wild-type populations. Finally, we reconstructed the signaling networks and compared them to the ground truth network of the Orton model. If both η and θ must be selected, we recommend evaluating how both parameters influence the residuals of the scCNR equations (Fig. S3C and D, available as supplementary data at *Bioinformatics* online), in order to identify an appropriate balance between model complexity and residuals.

**Figure 3.**
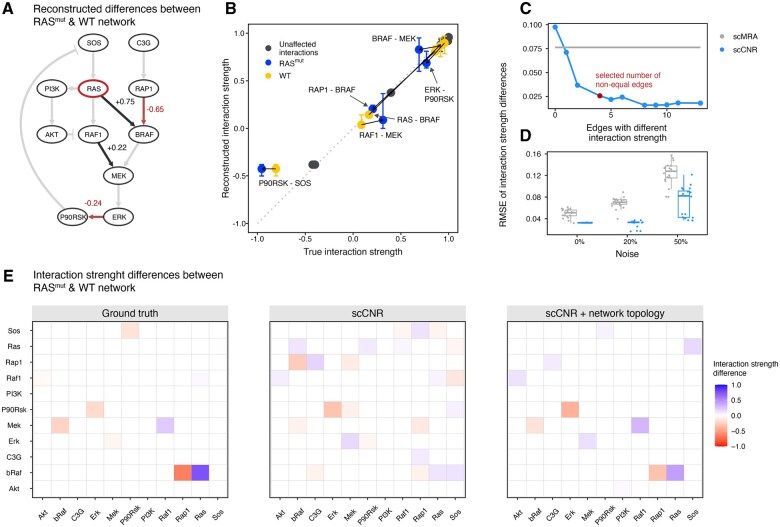
scCNR identifies signaling differences between cell populations. (A) Reconstructed differences between the RAS mutant and wild-type network. The mutated node is highlighted in red. Gray background edges indicate the true network topology. Black and red edges highlight the interactions that are stronger and weaker in the RAS mutant compared to the wild-type network, respectively. (B) Correlation between reconstructed and true interaction strengths averaged over 20 simulations. Blue points correspond to interaction strengths in the RAS mutant population, yellow in the wild-type population, and in gray points correspond to edges with the same interaction strength (difference below 0.1). Error bars visualize the range of reconstructed interaction strength values across simulations. Solid lines connect corresponding wild-type and mutant interaction strengths. (C) RMSE of interaction strength differences between the two populations for increasing numbers of population-specific interaction strengths. (D) RMSE for joined scCNR with four population-specific edges or separate scMRA reconstructions of the network, with various levels of noise. (E) Differences between the mutant and wild-type network for reconstructions with scCNR under intermediate noise conditions (noise = 10%). The values represent averages over five simulations. The left panel shows the ground truth differences, the middle panel shows the differences reconstructed using scCNR without prior network, and the right panel shows the differences reconstructed using scCNR guided by the prior network topology of the Orton model.

The simulations of the wild-type and RAS mutant cell population result in major differences upstream of BRAF, which scCNR recovered truthfully (Fig. S3A and Fig. S4A, available as supplementary data at *Bioinformatics* online). The BRAF mutant network, on the other hand, mainly differed downstream of BRAF within the BRAF-MEK-ERK-P90RSK cascade, which scCNR identified correctly as well (Figs S5A and 4D, available as supplementary data at *Bioinformatics* online). To quantitatively compare the scCNR reconstruction to the ground truth network, we reconstructed the signaling network for the wild-type and mutant cell population for 20 simulations as described above. The average reconstructed interaction strengths for both the RAS mutant and BRAF mutant cell populations correlated well with the true values for most edges (Fig. 3B and Fig. S5B, available as supplementary data at *Bioinformatics* online). With the restriction on detecting four population-specific interaction strengths (Fig. 3C), small interaction strength differences (such as BRAF-MEK), were not recovered in every simulation, as expected. However, edges with clear differences between the wild-type and mutant population were recovered well, such as RAP1-BRAF and RAS-BRAF (Fig. 3B).

### 3.6 Joint network reconstruction with scCNR improves performance over reconstruction with scMRA

In addition to highlighting the major differences between populations, we hypothesized that scCNR might improve network reconstruction by pooling cells from all populations into a single optimization problem, hence increasing the power. To test this hypothesis, we reconstructed the Orton network based on simulated wild-type and RAS mutant single-cell data of 500 cells in total, with 20% noise, and setting η to obtain networks with 13 edges. We quantified how well a combined reconstruction using scCNR recovers differences between mutant and wild-type networks, and compared this with two scMRA reconstructions where the wild-type and mutant populations are reconstructed independently. Neither scMRA nor scCNR was supplied with any prior knowledge of the network topology. We calculated the difference of interaction strengths between the two populations (wild-type and mutant) for both the ground truth and the reconstructed networks and compared these. Figure 3C shows the RMSE of the difference in interaction strengths as a function of the number of edges that have a different interaction strength between the two populations. The scCNR reconstructions (blue points) are consistently more accurate that the scMRA reconstructions (gray line), indicating the benefit of a joint network reconstruction. Increasing the number of differences above four results in little further improvement. With increasingly noisy input data, the increased performance of a joint reconstruction with scCNR becomes even more pronounced as seen in Fig. 3D and Fig. S4, available as supplementary data at *Bioinformatics* online. Together, this shows that by pooling information from cells of multiple populations scCNR can more accurately reconstruct relevant differences between populations as compared to independent scMRA reconstructions.

Real-world single-cell data are inherently noisy, making accurate reconstruction of complete network topologies challenging. However, in many experimental settings, the underlying network topology is at least partially known, and identifying significant quantitative differences in (known) interactions between cell populations might be both more feasible and relevant. To evaluate this, we added progressively increasing levels of noise to the data, which disrupted the correlation patterns between phospho-proteins (Fig. S6, available as supplementary data at *Bioinformatics* online). Under these high-noise conditions, scMRA could only recover 6 of the 13 true-positive edges with very weak interaction strengths, and differences between populations became indistinguishable by scCNR when no prior information about the network topology was provided (Fig. S7E, available as supplementary data at *Bioinformatics* online). However, when we reconstructed network differences using scCNR guided by the network topology, we were able to recover many of the true differences (albeit at reduced intensity) (Figs S7D and E and S8B and C, available as supplementary data at *Bioinformatics* online). Figure 3E demonstrates for simulations with the RAS-mutant and wild-type population that by constraining scCNR to known network edges, meaningful population differences can still be detected even when the data are noisy.

### 3.7 scCNR detects signaling difference in response to EGFR inhibition

Our results on the Orton model simulations show that scCNR recovers the main signaling differences between cell populations, especially when guided by prior network knowledge. Considering real use-cases, we intend scCNR to be used for well-characterized signaling networks to study cell-state-specific signaling or different drug perturbation effects in the network. Therefore, to further demonstrate the utility of scCNR, we applied the method to previously unpublished experimental single-cell measurements of primary human keratinocytes exposed to EGFR inhibitors or vehicle treatment. The purpose of the treatment was to quantify how prolonged EGFR inhibition affects signal transduction. Although the EGFR pathway has been well studied, how prolonged exposure to inhibitors alters signal transduction within the network is still an open question to which much attention is devoted (Klinger *et al.* 2013, Bosdriesz *et al.* 2018). In this experiment, we measured the abundance of 70 phosphorylated and total proteins of 282 cells using single-cell ID-seq (Eijl *et al.* 2018), involving 31 key nodes of the EGFR pathway, after treatment with an EGFR inhibitor (AG1478) or vehicle control (DMSO) for 48 h.

As expected, EGFR inhibition induced a reduction in phosphoprotein abundance of proteins downstream of EGFR, including RPS6 and AKT (Fig. S9A, available as supplementary data at *Bioinformatics* online) (Fan *et al.* 2009, Phuchareon *et al.* 2015). We detect reduced levels of phospho-RB after treatment, while CDK4 and Cycline-E stayed active, which marks an arrest of cells in the early cell-cycle stage (Fig. S9A, available as supplementary data at *Bioinformatics* online). Furthermore, EGFR inhibition pushes cells into a differentiated cell state, which is marked by a decrease in ITGB1. We also detect an increase of BMPR and it has been shown that BMP signaling goes up during differentiation (Eijl *et al.* 2018). EGFR inhibition additionally raises the level of phosphorylated p38, which has been linked to keratinocyte differentiation and cell-cycle arrest (Efimova *et al.* 1998, Adhikary *et al.* 2010, Connelly *et al.* 2011, Saha *et al.* 2014). Next to the increased phosphorylation of p38, rising levels of phosphorylated JNK have been reported for keratinocytes after EGFR-inhibitor treatment (Lu *et al.* 2011). Together, this demonstrates that the data contain biologically meaningful signal, and so we continued to model the underlying signaling differences between the two cell populations.

**Figure 4.**
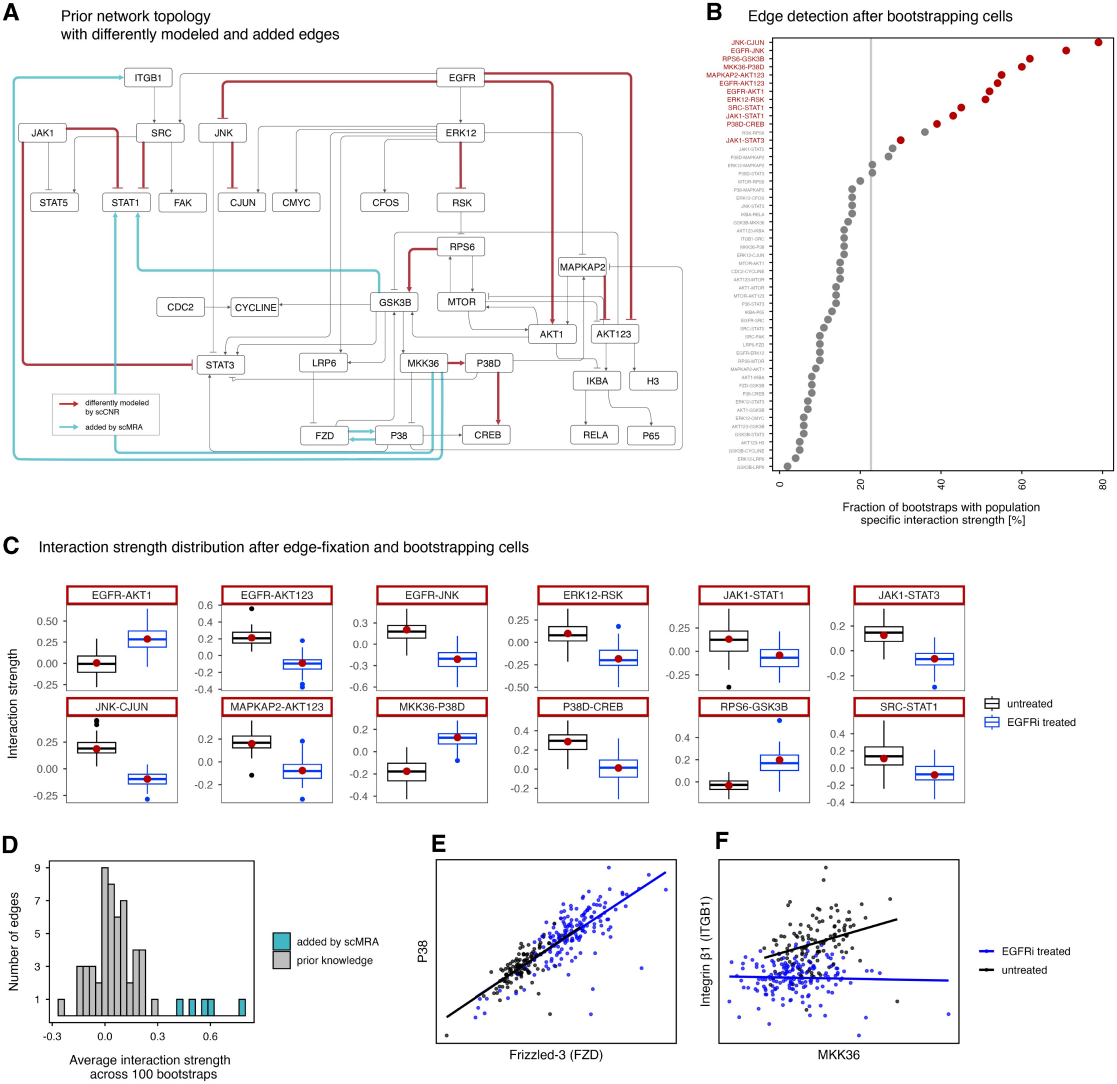
Analysis of signaling differences between untreated and EGFR-inhibitor-treated keratinocytes. (A) Prior knowledge network depicting reconstructed edges that differ between EGFR-inhibitor-treated and untreated cells in red and new edges added by scMRA in turquoise. (B) Bootstrapping analysis to detect edges that are consistently identified to have population-specific strengths. After every bootstrap, scCNR identified 12 population-specific interaction strengths and we counted their occurrence within all bootstrapped runs. Significant edges from A are highlighted in red. The vertical line corresponds to the theoretical expectation of randomly selecting an edge to have a population-specific interaction strength. (C) Distributions of interaction strengths from 100 bootstrapping runs, for the untreated and EGFR inhibitor-treated cells. The red dot indicates the recovered interaction strength from the full dataset, as depicted in A. (D) Distribution of the average interaction strengths for edges in the prior knowledge topology and the five edges added by scMRA. Average interaction strengths were obtained by fixing the prior knowledge network extended with the new edges, bootstrapping untreated cells 100 times and repeatedly inferring interaction strengths by scMRA. (E) Correlation between Frizzled-3 and P38, an edge that was repeatedly added by scMRA across 100 bootstraps and (F) the correlation between MKK36 and ITGB1—an edge that is modeled to be population-specific when re-running scCNR on the prior knowledge topology with the five new edges.

The average correlation between interacting phosphoproteins downstream of EGFR was below 0.1 and in simulations based on the Orton model, we observed that the underlying network topology could no longer be reliably reconstructed once correlations dropped below 0.2 (Figs S7 and S8, available as supplementary data at *Bioinformatics* online). Moreover, the inferred interaction strengths between well-established protein interactions were highly variable and frequently centered around zero, suggesting an intrinsically weak signal for network inference (Fig. S10, available as supplementary data at *Bioinformatics* online). Nevertheless, our simulations showed that guiding scCNR with the known network topology resulted in successful recovery of meaningful differences between cell populations even under high noise conditions (Fig. 3E). We therefore applied scCNR using a prior network topology consisting of canonical protein interactions derived from PhosphoSitePlus to identify differences of the EGFR signaling pathway between the untreated and EGFR-inhibitor-treated population (Hornbeck *et al.* 2015). We fixed edges of the prior network topology (by setting the corresponding indicator constraints to 1) and set the θ-parameter to identify 12 population-specific interaction strengths (Fig. 4A) to balance model complexity and model fit (Fig. S9B, available as supplementary data at *Bioinformatics* online). To assess the statistical significance of the differences in interaction strengths between the treated and untreated populations identified by scCNR, we permuted the population labels and reconstructed the interaction strengths 100 times, while fixing which of the interaction strengths can be population specific from the unpermuted solution. In this null model, we expect that the interaction strengths do not differ between populations. We calculated the empirical *P*-value as the fraction of permutations that led to a more extreme interaction strength difference than the ones obtained from the unpermuted network reconstruction. At a 20% false positive rate, all 12 population-specific interaction strengths proposed by scCNR had significant differences between populations (Fig. 4A). As expected from an inhibition of EGFR, we predominantly recover differences directly linked to that node. Three of five outgoing edges from EGFR differ in interaction-strength between the cell populations (Fig. S9C, available as supplementary data at *Bioinformatics* online).

Next, we conducted a bootstrapping analysis to verify that the selection of edges is not primarily driven by outlier cells. We subsampled cells with replacement from both populations, repeated the network reconstruction 100 times, and counted for each edge how often it was identified to be population specific. Edges around EGFR consistently show up in the reconstruction, supporting the fact that these edges are truly different between the cell populations. Moreover, all differentially recovered edges are far above the theoretical threshold for the occurrence of a randomly selected edge (Fig. 4B).

To investigate how reliable differences in interaction strength are *quantitatively*, we performed a bootstrapping analysis where we set all interaction-strength differences to zero except the ones for edges that were identified to differ in strength between populations, allowed only these edges to differ between populations, bootstrapped cells from both populations, and re-ran scCNR to recover new interaction strengths for these edges. These 100 bootstraps provided us with distributions of interaction strengths for both populations (Fig. 4C). Most interaction strengths showed clear differences between the cell populations.

Among the three population-specific edges downstream of EGFR, two decrease in interaction strength following inhibition. Also edges further downstream of EGFR decrease in interaction strength upon EGFR inhibition, such as ERK12-RSK, JNK-CJUN, MAPKAP2-AKT123, or SRC-STAT1. From this, we conclude that despite the limitations imposed by the relatively small sample size, scCNR is able to detect significant and biologically meaningful differences in signal transduction between populations.

Although high levels of noise in the data may hinder the recovery of the original network topology, scMRA can still add new data-driven edges to the prior network that can contain meaningful biological information, e.g. indirect regulatory connections between proteins that function within the same signaling cascade. To this end, we applied scMRA to the untreated cells, setting η such that five new edges are added to the network. The resulting interactions are among the strongest in the new network (Fig. 4D) and are repeatedly recovered when bootstrapping untreated cells and repeating the analysis (Fig. S11, available as supplementary data at *Bioinformatics* online).

Notably, the interaction between Frizzled-3 and p38 is consistently added to the network and is supported by a strong correlation between the associated proteins (Fig. 4E). Although no direct physical interaction between these two proteins has been reported, Frizzled-3 functions as a receptor in the Wnt pathway, which is known to modulate p38 activity (Ehyai *et al.* 2016). Another recurrent edge connects ITGB1 and MKK3/6. ITGB1 is a key regulator of MAPK signaling, although the interaction has been described predominantly in the reverse direction (Mainiero *et al.* 2000). Interestingly, scCNR additionally models this edge as population-specific: its strength decreases to almost zero upon EGFR inhibition (Fig. 4F), consistent with the well-established cross-talk between ITGB1 and EGFR (Su *et al.* 2024). While the inferred direction of the ITGB1–MKK3/6 edge and the bidirectional connection between Frizzled-3 and p38 likely reflect the limitations of inferring causal directionality when total protein or perturbation data is missing, these edges remain biologically plausible. We also identified an interaction between GSK3β and STAT1, which is consistent with prior evidence showing that GSK3β facilitates STAT1 activation downstream of cytokine and stress signaling, potentially through indirect regulation of upstream phosphatases such as SHP2 (Tsai *et al.* 2009). These newly added edges correspond to the strongest, most consistent relationships present in the data. While most likely not direct interactions, they capture robust patterns of co-regulation that best explain the observed signaling behavior.

## 4 Discussion

How signal transduction differs between cell states, in disease, or upon treatment, plays a fundamental role in cell biology and has potential clinical implications. Reconstructing signaling networks has long been limited to bulk data, but recent advances in single-cell (phospho-)protein measurement technologies allow us to take advantage of many data points to study signaling networks on the single-cell level. To this end, we developed scMRA and scCNR, which exploit the stochastic variability of protein abundances between cells as natural “perturbation experiments.” By penalizing network complexity and, in the case of scCNR, the differences in signaling between cell population, core signaling networks, and their most relevant differences are found. Additionally, prior network information can easily be integrated and can improve network reconstruction S2. We extensively validated the methods on simulated data and showed that scMRA works well with only 500 cells and low noise and that scCNR can accurately recover signaling differences even in high-noise scenarios when guided by a prior network topology. As a real-world validation, we treated cells either with an EGFR inhibitor or with a vehicle control, and recovered biologically meaningful and expected signaling differences between the treated and untreated cell populations. Although *de novo* network reconstruction relies on high signal strength and may currently be impractical, scCNR remains effective in detecting meaningful differences in signaling, especially when informed by prior network structure. In addition, scMRA can infer additional, data-driven edges that may reveal meaningful signaling interactions.

Our findings underscore the method’s capacity to identify relevant signaling differences, but it is essential to also acknowledge the limitations. While scCNR explains phosphoprotein counts for every cell individually, it does rely on (prior) clustering of cells into discrete groups to infer population-specific network parameters. Both scMRA and scCNR assume that the input cell populations are homogeneous and not further divisible into biologically meaningful subgroups. If a heterogeneous population containing distinct subpopulations is analyzed as a single group, the resulting network reconstruction will reflect an average signal, potentially masking subpopulation-specific interactions and yielding suboptimal results. One example is the inclusion of the pRB-high subpopulation within the untreated cells of the scID-seq dataset, as described in Supplementary Information 1.6, available as supplementary data at *Bioinformatics* online. Treating the pRB-high and pRB-low cells as a single population could introduce interactions driven by cluster differences rather than true biological interactions. We anticipate that future datasets with larger cell numbers will allow for more fine-grained subpopulation modeling.

In addition, some care needs to be taken in interpreting edges that the model proposes, as these might be indirect and mediated through unobserved nodes. Therefore, we expect the scCNR results to typically serve as a valuable foundation for generating hypotheses regarding mechanistic interactions, which can subsequently be subjected to further in-depth exploration. Future progress in single-cell protein measurement techniques will enhance the detection of cell-to-cell variability and will improve network inference with scCNR.

Similar to MRA and CNR, our method describes changes in active protein as a linear function of changes in its total abundance and upstream active protein. The methods therefore assume locally linear protein interactions, which might not hold true for cells that vary significantly from its population mean. However, since for most use cases cells will have been assigned to biologically meaningful populations (e.g. through prior clustering), we expect variation within these populations to be well described by a linearization around the population mean.

In contrast to the classical MRA and CNR formulation, the scMRA and scCNR optimization problems are typically over-determined since there are many more linear equations—one for every cell and every protein—than possible edges. Nonetheless, the computational complexity of the optimization problem increases exponentially with the number of nodes in the network. However, biological networks are often sparsely connected, and sparse solutions can generally be found efficiently. For instance, the Orton model can be solved in under 20 s for 500 cells with 20% noise and 13 edges on a standard laptop. The number of nodes in the network and the number of edges and population-specific interaction strengths influence the search space and therefore the run time. In cases where many interactions have to be inferred, the run time can be reduced drastically by incorporating prior network information or by setting hyperparameter settings that limit the number of model parameters that need to be inferred. We propose to use scCNR as a tool to get further insight into pathways with a known backbone topology, rather than recovering novel networks with hundreds of nodes *ab initio*. A typical use case would be to unravel network variation between cell states, where scCNR can add novel connections to an existing backbone topology.

In conclusion, scCNR recovers relevant differences in signaling between cell populations and we envision scCNR to be mainly used for signaling networks of specific interest, where the interacting nodes are known and the focus is on recovering the differences in interactions between populations, like cell-state dependent treatment effects, resistance mechanisms or signaling differences between treatment strategies. In this way, scCNR will further our understanding of signaling networks in diverse biological settings, especially by shedding light on clinically relevant signaling differences.

## Supplementary Material

btaf675_Supplementary_Data

## Software availability

The code used for scCNR in this study has been deposited on Zenodo https://doi.org/10.5281/zenodo.17600937 and is also available as a Python module at https://github.com/ibivu/scmra. Additionally, data and code to reproduce all figures is available at https://github.com/tstohn/scmra_analysis.
