# Supplementary material for: Reconstructing and comparing signal transduction networks from single-cell protein quantification data
Source: Bioinformatics. 2025 Dec 23;42(1):btaf675. doi: 10.1093/bioinformatics/btaf675 (PMC12797212; doi:10.1093/bioinformatics/btaf675)
Supplement: btaf675_Supplementary_Data [file btaf675_supplementary_data.pdf]

Supplementary Information for: Reconstructing and comparing signal transduction networks from single-cell protein quantification data

S.1 Supplementary Methods

S.1.1 Evaluating scMRA and scCNR

For the analyses presented in Fig. 2 E and Fig. 3, to compare networks with the same number of edges, we reconstructed networks iteratively and varied the η parameter until we reached a network of 13 edges. 13 edges also show a major reduction in the mean squared error of the objective function while keeping a sparse network topology (Fig. S1B). For the evaluation of scCNR in Fig. 2 and Fig. S5 we followed the same procedure and compared only reconstructed networks with 13 edges. Balancing the model complexity and the reduction in the mean squared error resulted in 4 population-specific interaction strengths for the scCNR analysis of the RAS mutant population as well as for the BRAF mutant population (Fig. S3). To evaluate how well scCNR detects differences we calculated the RMSE of the interactions strenghts differences in Fig. 3C,D. scCNR results in population-specific interaction strenghts that share the same network topology. We calculated the difference for all edges in the network between the two populations. We did the same for the ground truth data of the wild type and mutant simulations. To compare the true with the recovered differences we calculated the RMSE between these two sets. The increased performance of scCNR to identify signaling differences is exemplified in network reconstructions based on very noisy (50%) input data (Fig. S4). We performed reconstructions based on 20 simulation runs, and calculated the average interaction strength differences of these 20 reconstructions. The two main interaction differences for the comparison of the RAS mutant and wild-type network, RAS-BRAF and RAP1-BRAF, are discovered in both the scCNR and scMRA approach. For scCNR we find two solutions across the 20 simulations (bimodal RMSE of interaction strength differences (Fig. 3D))). One solution models the network correctly with edges from RAS and BRAF, while the other solution identifies wrong interactions from SOS, C3G. However, scMRA detects many false positive interaction differences, such as RAS-SOS (present in all 20 simulations), RAP1-C3G (present in 70% of the simulations) and P90RSK-SOS (present in 90%) with a wrong sign of the interaction strength difference.

S.1.2 Cell culture and reagents

Primary pooled human epidermal stem cells derived from foreskin were obtained from Lonza. Cells were cultured and expanded as previously reported (Gandarillas and Watt, 1997). Briefly, cells were cultured on a feeder layer of J2-3T3 cells in FAD medium (Ham's F12 medium/Dulbecco's modified Eagle medium (DMEM) (1:3) supplemented with 10% batch

tested fetal calf serum (FCS) and a cocktail of 0.5 µg/ml of hydrocortisone, 5 µg/ml of insulin, 0.1 nM cholera enterotoxin, and 10 ng/ml of epidermal growth factor) supplemented with Rock inhibitor (Y-27632, 10 µM). J2-3T3 cells were cultured in DMEM containing 10% bovine serum and inactivated with Mitomycin C (SCBT) upon seeding the epidermal stem cells. For experiments epidermal stem cells were transferred to Keratinocyte Serum Free Medium (KSFM) supplemented with 0.2 ng/ml Epidermal Growth Factor and 30 µg/ml bovine pituitary extract from Life Technology until 70% confluent. Cells were treated with AG1478 (10 µM, Calbiochem) or vehicle (DMSO) for 48 hours prior to harvesting and fixation. All media were supplemented with 1% penicillin/streptomycin antibiotics.

S.1.3 Antibody conjugation with dsDNA barcodes

Antibodies and dsDNA were functionalized and conjugated as described (Buggenum *et al.*, 2016). The antibody panel was used as in (Eijl *et al.*, 2018) and details are provided in supplemental Table 1 of this paper. In short, antibodies were functionalized with NHS-s-s-PEG4-tetrazine (Jena Bioscience) in a ratio of 1:10 in 50 mM borate buffered Saline pH 8.4 (150 mM NaCl). Then, N3-dsDNA was produced and functionalized with DBCO-PEG12-TCO (Jena Bioscience) in a ratio of 1:25 (oligo list). Finally, purified functionalized antibodies were conjugated to purified functionalized DNA by 4-hour incubation at room temperature in borate buffered saline pH 8.4 in a ratio of 4:1 respectively. The reaction was quenched with an excess of 3,6-diphenyl tetrazine. The conjugation efficiency and quality were checked on an agarose gel, confirming that a substantial amount of DNA conjugated with the antibody. Ultimately, conjugates were equally pooled for staining's in scID-seq.

S.1.4 Immunostaining and single-cell sorting

The scID-seq workflow was performed essentially as described in (Eijl *et al.*, 2018), In brief, cells ($> 3x10^6$) were harvested with trypsin and cross-linked in suspension by incubating for 10 minutes with 4% paraformaldehyde (PFA) in PBS following a quenching step of 5 minutes with 125 mM Glycine in PBS. Removal of PFA and Glycine occurred through washing twice with wash buffer (0.1x Pierce™ Protein-Free Blocking Buffer from Thermo in PBS). Then, cells were blocked in 500µl blocking buffer (0.5x 0.1x Pierce™ Protein-Free Blocking Buffer, 200 µg/ml boiled salmon sperm DNA, 0.1% Triton-X 100, in PBS) at room temperature for 30-60 min. Staining with the conjugate mix occurred overnight at 4℃ in 500 µl blocking buffer and washed 3x in 5ml wash buffer. Individual cells were sorted with the BD FACSAria SORP flow cytometer (BD biosciences) in 96 well PCR plates containing 1µl release buffer (10 mM DTT in 15mM Tris, pH 8.8) and 7µl Vapor-lock (Qiagen). Plates were stored at -20℃ until use.

S.1.5 Barcoding and library preparation for next generation sequencing

For the library preparation 3 PCR steps were performed to amplify the antibody barcodes and to add barcodes specific for the well and the plate of each cell. The barcoding occurred with the same sequences used in ID-seq (Buggenum *et al.*, 2016). For the first PCR step 15 cycles were run after adding to each well a 4 µl reaction mix containing the Herculase II Fusion DNA Polymerase (Agilent), dNTPs, 5x Herculase buffer and 0.1µM amplification primers (Forward `5'-CACGACGCTCTTCCGATCT-3'`, Reverse `5'-TCGCTTATCTGTTGACTGAT-3'`). Directly after the first PCR step, 5 extra cycles were run after adding 1 µl mix containing Herculase buffer 0.2 µM forward amplification primer and 0.2 µM reverse well barcoding primer. Then all material was pooled per plate, Vapor-lock was removed and a clean-up was performed with the QIAquick PCR Purification Kit, an EXO1 treatment to degrade remaining primers followed by another purification. Another 5 cycles were run in PCR 3 with a 20 µl reaction containing pooled and purified plate sample and 0.1 µM plate barcoding primers (Fw_long `5'-AATGATACGGCGACCACCG AGATCTACACTCTTTCCCTACACGACGCTCTTCCGATCT-3'` and specific plate reverse). After repeating the clean-up, the libraries were checked on agarose gel and with the Bioanalyzer (Agilent) to confirm the size of the DNA fragments (expected size around 185 bp).

S.1.6 scID-seq data analysis

Sequence data from the NextSeq500 (Illumina) was demultiplexed using bcl2fastq software (Illumina). Then, all reads were processed using our dedicated R-package (van Buggenum et al., 2018). In short, the sequencing reads were split using a common “anchor sequence” identifying the position of the UMI sequence, Barcode 1 (antibody specific) and Barcode 2 (well specific) sequence. After removing all duplicate reads, the number of UMI sequences were counted per barcode 1 and 2. Finally, barcode 1 (“antibody)” and barcode 2 (‘well’) sequences were matched to the corresponding. For scID-seq, a threshold was set based on the total UMI count per well difference between high- and low-quality cells or empty wells. Antibodies with a median of < 10 counts per cell were removed from the dataset. The data was normalized using TMM with the default A-values (Robinson and Oshlack, 2010). Since the dataset contained a defined panel of measured proteins we reduced the M-values to 5% to keep a sufficient set of proteins for the normalization. Differential abundance of phosphoproteins between the untreated and EGFR inhibitor treated cell population was calculated with DESeq2.

The signaling differences between the untreated and EGFR-inhibitor treated cell populations were reconstructed using scCNR. The data displayed considerable heterogeneity, especially the cell cycle marker phospho-RB had a bimodal distribution separating cells into an actively cycling and non-cycling subgroup for the untreated population. Since the number of cells was limited (164 untreated and 118 treated cells) we nevertheless did not further divide

the untreated group into sub-populations. Outliers were removed separately for each population by removing cells with a protein abundance difference from the population mean that exceeded three standard deviations. This reduced the total cell counts to 117 untreated and 95 treated cells and resulted in recovered network differences that appeared stable towards outliers (Fig. 4).

The EGFR network topology is well studied and we started the analysis with a prior network. To construct it, we added canonical interactions as well as known interactions from PhoshoSitePlus® to the network (Hornbeck *et al.*, 2015). Proteins without canonical interactions were removed for the scCNR/scMRA analyses. We then estimated the penalty for the number of non-equal edges (θ) to reconstruct a network with 12 different edges. Phospho-RB follows a bimodal distribution in the untreated cell population and represents cells in different cell-cycle stages. Including phospho-RB in the analysis resulted in population-specific edges around this node due to a second phospho-RB high cell population within the untreated population. While the edge CDC2-RB was significant with a false discovery rate below 10%, using bimodal features to describe signaling in one cell population defies the purpose of describing cell-state specific signaling networks. Therefore, we decided to exclude phospho-RB from this analysis.

The permutation analysis to assess the statistical significance of interaction strength differences depicted in (Fig. 4A) was performed by randomly shuffling cell state labels. The network topology and the interactions that differ were fixed by constraining all indicator variables to be the value they had in the original model. Subsequently, scCNR was re-run 1000 times to obtain the interaction strengths for the permuted data. This way we obtain a null-distribution of interaction-strength-deviations from the population mean (Fig. S9C). The bootstrap analysis to assess the robustness of interaction-strength-difference detection in Fig. 4B was performed by randomly sampling 212 cells (i.e. the original number of cells) with replacement, and rerunning scCNR with its original parameters. This procedure was repeated 100 times. From this, the fraction of bootstraps in which an edge is predicted to be different can be obtained. In Fig. 4C we fixed the interaction strengths differences found in Fig. 4A by enforcing the corresponding indicator constraints in the scCNR model. We then performed the bootstrapping analysis similarly as above by sampling 212 cells with replacement and repeating the bootstrapping 1000 times. From this we obtained distributions of interaction strengths in both populations for the 12 population-specific interaction strengths. To add edges to this prior network topology we ran scMRA on only the untreated cells and set the η parameter so that scMRA recovered five additional edges. To obtain an estimate of the interaction strength distributions we ran 100 bootstraps with the new fixed network topology (Fig. 4D). After adding these five edges to the network topology we re-ran scCNR to see if any of these edges would now be modeled as a population-specific interaction, and found that this was the case for two edges: ITGB1-MKK36 and STAT1-GSK3B. We calculated the significance of these two population-specific edges by repeating the above described permutation analysis (Fig. S9D).

S.2 Derivation of the single-cell MRA equation

Here, we will derive the main equations underlying single cell MRA and single cell CNR. Underlying these derivations, there are three main biologically motivated assumptions. These assumptions are motivated and illustrated with a toy example in section S.3 These assumptions are:

1. Proteins show stochastic fluctuations in their abundances (even within a cell state). These stochastic fluctuations will in turn affect the abundance of the phosphorylated level of the protein. Technically, this implies that the *partial* derivative of the abundance of a phospho-protein with respect to its corresponding total protein is non-zero.
2. The total abundances of a protein does not have a *direct* effect on the abundance of other phospho-proteins. Technically, this implies that the partial derivative of a phospho-protein with respect to other total proteins is zero. Note change total protein abundances might affect different phospho-proteins *indirectly*, i.e. through changes in the abundance of the corresponding phospho-protein.
3. The timescale at which total protein abundances fluctuate is much slower that of phospho-proteins. Technically, this implies that the system is in quasi-steady state.

To derive the scMRA equations, we now consider a dataset of N_c cells, in which we have measured the activity x_i and total abundance x_i^{tot} of N_n proteins, which are the nodes of the signaling network we want to reconstruction. Optionally, a set of N_p perturbations by, e.g., small molecule inhibitors was performed. The activity of node i in cell a, $x_{i,a}$ can be described as the population average activity of node i, $\langle x_i \rangle$ plus some deviation $\Delta_a x_i$.

$$x_{i,a} = \langle x_i \rangle + \Delta_a x_i \tag{S1}$$

and similarly for the total amount of protein $x_i^{\text{tot}} = \langle x_i^{\text{tot}} \rangle + \Delta_a x_i^{\text{tot}}$.

Assumptions 1 and 2 above imply that the dynamics $x_{i,a}$ are a function of it's total abundance and the activity of the other proteins in the network (denoted by $\mathbf{x}_a$). The quasi steady state assumption furthermore implies that the time derivative of $x_{i,a}$ vanishes. Together, this implies:

$$\frac{\mathrm{d}x_{i,a}}{\mathrm{d}t} = f_i\left(\mathbf{x}_a, x_{i,a}^{\text{tot}}, \mathbf{p}\right) \equiv f_{i,a} \approx 0 \quad \forall a, i. \tag{S2}$$

where $\mathbf{p}$ is a vector of parameters describing the system. Note that if each cell individually is in a quasi steady state, the bulk average of all cells also needs to be:

$$\frac{\mathrm{d}\langle x_i \rangle}{\mathrm{d}t} = f_i\left(\langle \mathbf{x} \rangle, \langle x_i^{\text{tot}} \rangle, \mathbf{p}\right) \equiv \langle f_i \rangle = 0 \quad \forall i. \tag{S3}$$

Perturbations to the network, such treatment with small molecule inhibitors, can be modded as a perturbation to the parameters in the unperturbed reference condition $\mathbf{p_0}$, i.e. $\mathbf{p} = \mathbf{p}_0 + \Delta\mathbf{p}$

The first order Taylor expansion of $f_{i,a}$ around $\langle x_i \rangle$ is given by:

$$f_{i,a} \approx \langle f_i \rangle + \sum_{j=1}^{N_n} \frac{\partial f_i}{\partial x_j} \cdot \Delta_a x_j + \frac{\partial f_i}{\partial x_i^{\text{tot}}} \cdot \Delta_a x_i^{\text{tot}} + \sum_{m=1}^{N_p} \frac{\partial f_i}{\partial p_m} \cdot \Delta p_m, \tag{S4}$$

where we made use of assumption 2, i.e. that $\frac{\partial f_i}{\partial x_j^{\text{tot}}} = 0$ if $i \neq j$. Also note that $\frac{\partial f_i}{\partial p_m} \neq 0$ only for those perturbations p_m that *directly* affect node x_i, such as a targeted inhibitor and its kinase target. Multiple targets for the perturbations p_m can be accounted for, as long as the targets are known.

Dividing everything by $\langle x_i \rangle \frac{\partial f_i}{\partial x_i}$ and using that $f_{i,a}$ and $\langle f_i \rangle$ are 0 this gives

$$\begin{aligned} 0 \approx & \frac{\Delta_a x_i}{\langle x_i \rangle} + \sum_{j \neq i} \frac{\langle x_j \rangle}{\langle x_i \rangle} \left(\frac{\partial f_i}{\partial x_j} / \frac{\partial f_i}{\partial x_i} \right) \cdot \frac{\Delta_a x_j}{\langle x_j \rangle} + \\ & \frac{\langle x_i^{\text{tot}} \rangle}{\langle x_i \rangle} \left(\frac{\partial f_i}{\partial x_i^{\text{tot}}} / \frac{\partial f_i}{\partial x_i} \right) \cdot \frac{\Delta_a x_i^{\text{tot}}}{\langle x_i^{\text{tot}} \rangle} + \\ & \frac{p_m}{\langle x_i \rangle} \left(\frac{\partial f_i}{\partial p_m} / \frac{\partial f_i}{\partial x_i} \right) \cdot \frac{\Delta p_m}{p_m}. \end{aligned} \tag{S5}$$

the implicit function theorem states that

$$\frac{\partial f_i}{\partial x_j} / \frac{\partial f_i}{\partial x_i} = -\frac{\partial x_i}{\partial x_j}, \quad \frac{\partial f_i}{\partial x_i^{\text{tot}}} / \frac{\partial f i}{\partial x i} = -\frac{\partial x_i}{\partial x_i^{\text{tot}}}, \text{ and } \frac{\partial f_i}{\partial p_m} / \frac{\partial f_i}{\partial x_i} = -\frac{\partial x_i}{\partial p_m},$$

so that we end up with the equation:

$$R_{i,a} \approx \sum_{j \neq i} r_{ij} \cdot R_{j,a} + s_i \cdot R_{i,a}^{\text{tot}} + \sum_m p_{im} \quad \forall a, i. \tag{S6}$$

where:

$$r_{ij} \equiv \frac{\langle x_j \rangle}{\langle x_i \rangle} \frac{\partial x_i}{\partial x_j} \tag{S7}$$

is the interaction strength between node j and node i, with $r_{ii} \equiv -1$.

$$s_i \equiv \frac{\langle x_i^{\text{tot}} \rangle}{\langle x_i \rangle} \frac{\partial x_i}{\partial x_i^{\text{tot}}}, \tag{S8}$$

is the sensitivity of x_i to changes in it's total abundance,

$$p_{im} \equiv \frac{p_m}{\langle x_i \rangle} \frac{\partial x_i}{\partial p_m} \frac{\Delta p_m}{p_m} \tag{S9}$$

is the direct effect that perturbation m has on the activity of node i,

$$R_{i,a} \equiv \frac{\Delta_a x_i}{\langle x_i \rangle} \tag{S10}$$

is the deviation of the activity of node i from the population average and

$$R_{i,a}^{\text{tot}} \equiv \frac{\Delta_a x_i^{\text{tot}}}{\langle x_i^{\text{tot}} \rangle} \tag{S11}$$

is the deviation of the total abundance of protein i from the population average.

In matrix-form, equation S6 reads:

$$\mathbf{r} \cdot \mathbf{R} + \mathbf{s} \cdot \mathbf{R}^{\text{tot}} + \mathbf{p} \approx 0 \tag{S12}$$

r_{ij}, s_i and $p_{i,m}$ are the unknown quantities that we want to estimate based on the measurements $R_{i,a}$ and $R^{\text{tot}}_{i,a}$. For this, we have system of $N_n \times N_c$ equations (one for each cell for each node). In theory, there are $N_n \times N_n + N_n + N_n \times N_p$ unknowns. However, in practice we will know for the vast majority of the $N_n \times N_p$ elements of p_{im} that they are 0, because most nodes are not affected by most inhibitors.

S.3 Assumptions underlying the derivation of single cell MRA

As stated above, there are three main assumptions underlying the derivation of equations S6

1. Fluctuations in total protein level affect the level of the corresponding active protein. Technically, this means:
$$\frac{\partial x_i}{\partial x_i^{\text{tot}}} \neq 0. \tag{S13}$$
2. Fluctuations in total protein levels do not *directly* affect the level of activity other active proteins. Technically, this means
$$\frac{\partial x_j}{\partial x_i^{\text{tot}}} = 0, \quad \forall i \neq j. \tag{S14}$$
3. The system is in (quasi)-steady state. This means $\frac{\mathrm{d}x_i}{\mathrm{d}t} \approx 0, \forall i$

Below, we will motivate and illustrate the biological rationale behind these with a simple toy model of a two-tired cascade, but all results readily generalize to more complex systems. Consider two protein-kinases, A and B[1] that are both described by a simple phosphorylation-dephosphorylation scheme, and where the phosphorylation of B is catalyzed by A (Scheme 1).

[1] On notation: subscript A and B refer to these two specific proteins, whereas i and j are used to refer to any protein in a network.

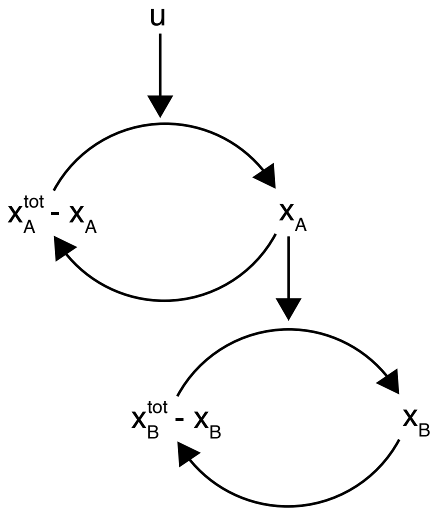

Scheme 1: Simple toy model: phosphorylation of protein B by protein A.

Using simple mass-action kinetics gives the following ordinary differential equations (ODEs) to describe this system

$$\frac{\mathrm{d}x_A}{\mathrm{d}t} = v_{+,A} - v_{-,A} = k_{cat,u} \cdot u \cdot (x_A^{\text{tot}} - x_A) - k_{phos,y} \cdot y \cdot x_A \tag{S15}$$

$$\frac{\mathrm{d}x_B}{\mathrm{d}t} = v_{+,B} - v_{-,B} = k_{cat,A} \cdot x_A \cdot (x_B^{\text{tot}} - x_B) - k_{phos,y} \cdot y \cdot x_B \tag{S16}$$

(S17)

where $v_{+,i}$ and $v_{-,i}$ are the rate of phosphorylation and dephosphorylation of protein i, respectively, u is an input to A, y is a phosphatase that dephosphorylation both A and B, and $k_{cat,i}$ is the catalytic rate constant of protein i. Solving the steady state for the equations above then gives (i.e. setting $\frac{\mathrm{d}x_A}{\mathrm{d}t}$ and $\frac{\mathrm{d}x_B}{\mathrm{d}t}$ to 0):

$$x_A = \frac{k_{cat,u} \cdot u}{k_{phos,y} \cdot y + k_{cat,u} \cdot u} \cdot x_A^{\text{tot}} \tag{S18}$$

$$x_B = \frac{k_{cat,A} \cdot x_A}{k_{phos,y} \cdot y + k_{cat,A} \cdot x_A} \cdot x_B^{\text{tot}} \tag{S19}$$

To consider the *direct* effect of a change in total protein abundance on the abundance of the corresponding phospho-protein, we consider what would happen to the abundance of the phosphoprotein if we could change the abundance of the total protein, while keeping everything else (e.g. all other phosphoproteins) in the network the same. Technically, this gives us the partial derivative of the steady state abundance of the phospho-protein with

respect to the total protein, i.e. $\frac{\partial x_i}{\partial x_i^{\text{tot}}}$. Calculating these gives:

$$\frac{\partial x_A}{\partial x_A^{\text{tot}}} = \frac{k_{cat,u} \cdot u}{k_{phos,y} \cdot y + k_{cat,u} \cdot u} \neq 0, \quad \text{and} \tag{S20}$$

$$\frac{\partial x_B}{\partial x_B^{\text{tot}}} = \frac{k_{cat,A} \cdot x_A}{k_{phos,y} \cdot y + k_{cat,A} \cdot x_A} \neq 0 \tag{S21}$$

which shows that assumption 1 is valid. In other words, if we have a natural perturbation experiment where x_i^{tot} is "perturbed", this will have a "direct" effect on x_i (in addition to potential indirect effects mediated through feed-backs). In this cases, the *fraction* of protein that is phosphorylated remains constant for both A and B (hence, $s_i \equiv \frac{x_i^{\text{tot}}}{x_i} \frac{\partial x_i}{\partial x_i^{\text{tot}}} = 1$ for A and B).

Our second assumptions states that the *direct* effect of changes x_i^{tot} do not affect x_j if $j \neq i$. Here, by direct effect we mean the effect of $x^{\text{tot}})i$ that is *not* mediated through changes in x_i. In our toy system, x_A does not depend on x_B at all, so this is trivially true. Furthermore, we have that

$$\frac{\partial x_B}{\partial x_A^{\text{tot}}} = \frac{\partial}{\partial x_A^{\text{tot}}} \left(\frac{k_{cat,A} \cdot x_A}{k_{phos,y} \cdot y + k_{cat,A} \cdot x_A} \cdot x_B^{\text{tot}} \right) = 0 \tag{S22}$$

so we see that our second assumption is also valid. (Note that changes in x_A^{tot} do affect x_B *indirectly*, through changes in x_A).

Finally, we assume that our system is in quasi steady state. This implies that the timescales at which kinase phosphorylation and dephosphorylation occur are much faster than the timescales at which protein synthesis and dilution or degradation occur. In our toy model, we have not explicitly modeled the dynamics of x_A^{tot} and x_B^{tot}. This can, however, be straightforwardly incorporated by adding two ODEs $\frac{\mathrm{d}x_i^{\text{tot}}}{\mathrm{d}t} = v_{synth,i} - v_{deg,i} - \mu \cdot x_i^{\text{tot}}$ (for $i = A$ and $i = B$), where $v_{synth,i}$ is the total rate of x_i^{tot} synthesis (i.e. translation), $v_{deg,i}$ the total rate of degradation and $\mu \cdot x_i^{\text{tot}}$ the rate of dilution due to growth (with μ the specific growth rate). Mathematically, assumption 3 implies that typically $v+, i, v_{-,i} \gg v_{synth,i}, v_{deg_i}, \mu$. While this is not something we can derive from first principle, it is consistent with the literature . Changes in total protein abundance typically vary over the timescales of cell generations, i.e. days to weeks Sigal *et al.* (2006), whereas protein phosphorylation dynamics upon perturbations typically happen much faster. For instance, upon RTK-stimulation, ERK activity typically peaks after less then 5 minutes Ryu *et al.* (2018); Ahmed *et al.* (2014); Kim *et al.* (2023). Within these 5 minutes the signal needs have propagated throughout the whole network, placing the timescale of phosphorylation in signaling cascades in the seconds-minutes range

Together, these assumptions apply that if we can measure x_i^{tot} we can treat its variations as natural perturbation experiments. We have shown it here for a very simple system. In most cases the situation will be more complex, but the general principles will still hold: that we can expect the partial derivative of x_i with respect to x_i^{tot} to be non-zero, that with respect to x_j^{tot} to be zero (for $j \neq i$), and that protein phosphorylation dynamics are generally faster than protein synthesis.

S.4 Formulation of the MIQP optimization problems

S.4.1 single-cell Modular Response Analysis

Our aim is to find a network model that accurately describes the data, without being overly complex. To this end, similar as for "bulk" Comparative Network Reconstruction (Bosdriesz *et al.*, 2018), we formulate the problem as a Mixed Integer Quadratic Programming (MIQP) problem. We aim to solve Equation S6, while placing an L_0 penalty on the presence of an edge. Given N_n nodes in the network, N_c cells measured and for $i, j \in \{1, 2, \ldots, N_n\}$, $n \in \{1, 2, \ldots, N_p\}$ and $a \in \{1, 2, \ldots, N_c\}$, the MIQP problem then reads as follows:

$$
\begin{aligned}
\text{minimize} : & \sum_{a,i,j} \left[(1-\eta) \cdot \epsilon_{i,a}^2 + \eta \cdot I_{ij}^{\text{edge}} \right] \\
\text{subject to} : & \sum_{j=1}^{N_n} r_{ij} \cdot R_{j,a} + s_i \cdot R_{i,a}^{\text{tot}} + \sum_{m=1}^{N_p} p_{im} = \epsilon_{i,a} \quad \forall a, i \\
& r_{ii} = -1 \\
& s_i > 0 \quad \forall i \\
& I_{ij}^{\text{edge}} = 0 \Rightarrow r_{ij} = 0 \quad \forall i, j \\
& I_{ij}^{\text{edge}} \in \{0, 1\}
\end{aligned}
$$

where I_{ij}^{edge} are so-called indicator constraints indicating the presence or absence of an edge from node j to node i, which are used to implement the L_0 penalty. The hyperparameter η is used to tune the complexity of the model. We also make the assumption that an increase in total protein abundance increases node activity: $s_i > 0$.

In some cases, for some nodes only the activity but not their total abundance is measured, i.e. $R_{i,a}^{\text{tot}}$ is not measured for protein i. In that situation we cannot estimate s_i. The most straightforward solution would be to ignore the $s_i \cdot R_{i,a}^{\text{tot}}$ term. As a results, the residuals for these nodes would be higher, on average. To account for this, we can weight the residuals of "complete" and "incomplete" nodes differently to balance their contributions. Specifically, this requires finding a factor α (between 0 and 1) such that $\alpha \cdot \langle \epsilon_{\text{I}}^2 \rangle = \langle \epsilon_c^2 \rangle$ with the subscript referring to incomplete and complete nodes, respectively.

This requires estimating the contribution of the $s_i \cdot R_{i,a}^{\text{tot}}$-terms to explaining the response, which is not possible exactly. However, we can get an upper and lower bound, and take the mean to get a rough estimate of the residuals of nodes with missing total protein::

- For the upper bound, we assume there is no contribution from local responses r_{ij}. and protein activity is explained solely by its total protein. We can obtain this by calculating the residuals with $r_{ij} = 0$ (no edges in the network). This basically means regressing $R_{i,a}$ against $R_{i,a}^{\text{tot}}$. We calculate the upper bound of α as the ratio of average residuals for complete and incomplete nodes.

- For the lower bound, we assume there is a maximal contribution from local responses r_{ij}. We obtain this by not penalizing edges at all in the reconstruction, i.e. by setting $\eta = 0$, which results into a fully connected network. Like before we calculate the lower bound of α as the ratio of average residuals for complete and incomplete nodes.

In reality, the model reconstructs a network topology which neither has zero edges, nor that is fully connected. Therefore, to approximate a reasonable α we take the mean of the upper and the lower bound.

S.4.2 Formulation of single-cell Comparative Network Reconstruction

In many instances, we are interested in comparing the signaling networks between N_{cs} different cell populations ξ, with each cell state containing N_c^ξ $\xi \in \{1, \ldots, N_{cs}\}$ cells. Similar as for bulk Comparative Network Reconstruction (Bosdriesz *et al.*, 2018), the main idea is to formulate the MRA equation S6 on a per-cell population basis, and solve these simultaneously in an MIQP, while placing an L_0-penalty on quantitative differences between the networks. Note that in such a case, the deviations of total and phosphoprotein abundances should be calculated relative to the average of the cell population ξ, i.e. $x_{i,a} = \langle x_i^\xi \rangle + \Delta_a x_i$.

To that end, we can formulate Equation S.4.1 on a per-cell state basis.

$$R_{i,a} \approx \sum_{j \neq i} r_{ij}^\xi \cdot R_{j,a} + s_i^\xi \cdot R_{i,a}^{\text{tot}} + p_{im}^\xi \quad \forall a, i. \tag{S23}$$

where the ξ-superscript refers to the cell population. The variables than become:

$$r_{ij}^\xi \equiv \frac{\langle x_j^\xi \rangle}{\langle x_i^\xi \rangle} \frac{\partial x_i^\xi}{\partial x_j^\xi} \tag{S24}$$

$$s_i^\xi \equiv \frac{\langle x_i^{\text{tot},\xi} \rangle}{\langle x_i^\xi \rangle} \frac{\partial x_i^\xi}{\partial x_i^{\text{tot},\xi}} \tag{S25}$$

$$R_{i,a} \equiv \frac{\Delta_a x_i}{\langle x_i^\xi \rangle} \tag{S26}$$

$$R_{i,a}^{\text{tot}} \equiv \frac{\Delta_a x_i^{\text{tot}}}{\langle x_i^{\text{tot},\xi} \rangle} \tag{S27}$$

The full scCNR MIQP formulation becomes:

$$\begin{aligned}
\text{minimize}: & \sum_{a,i,j,m}\Big[(1-\eta)\cdot\epsilon_{i,a}^2+\eta\cdot I_{ij}^{\text{edge}}+\theta(I_{ij}^r+I_i^s+I_{im}^p\Big] & \forall a,i,j. \\
\text{subject to}: & \sum_{j=1}^{N_n} r_{ij}^{\xi}\cdot R_{j,a}+s_i^{\xi}\cdot R_{i,a}^{\text{tot}}+\sum_{m=1}^{N_p} p_{im}^{\xi}=\epsilon_{i,a} & \forall a,i \\
& r_{ii}^{\xi}=-1 & \\
& s_i^{\xi}>0 & \forall i \\
& I_{ij}^{\text{edge}}=0\Rightarrow r_{ij}=0 & \forall i,j \\
& I_{ij}^{r}=0\Rightarrow r_{ij}^{\xi}=\langle r_{ij}\rangle & \forall i,j \\
& I_{i}^{s}=0\Rightarrow s_{i}^{\xi}=\langle s_{i}\rangle & \forall i \\
& I_{im}^{p}=0\Rightarrow p_{im}^{\xi}=\langle p_{im}\rangle & \forall i,m \\
& I_{ij}^{\text{edge}},I_{ij}^r,I_i^s,I_{im}^p\in\{0,1\} &
\end{aligned}$$

where

$$\begin{aligned}
\langle r_{ij}\rangle &\equiv \frac{1}{N_{cs}}\sum_{\xi} r_{ij}^{\xi}, \\
\langle s_{i}\rangle &\equiv \frac{1}{N_{cs}}\sum_{\xi} s_{i}^{\xi}, \text{ and} \\
\langle p_{im}\rangle &\equiv \frac{1}{N_{cs}}\sum_{\xi} p_{im}^{\xi}
\end{aligned} \tag{S28}$$

S.5 Supplementary Figures

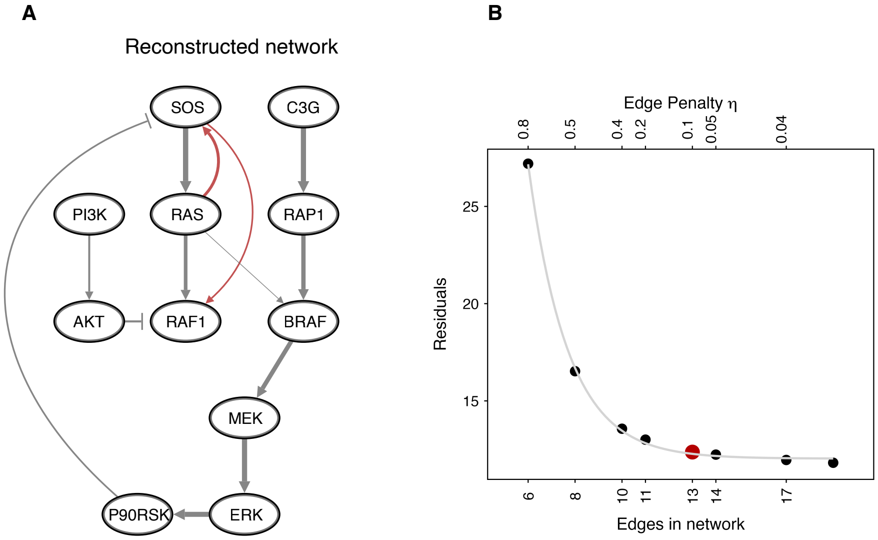

Figure S1: Exemplary scMRA network reconstruction with 13 edges. (**A**) An example of a reconstructed network for a simulation of the Orton Model as in (Fig. 2C) with 1000 cells and 20% noise. (**B**) To balance a good network reconstruction and a simple network topology (focussing on the main edges) we recommend to pick an η at the "elbow" of the curve for the final reconstruction, where the residuals of the scMRA equations show minimal improvement with increasing network complexity. Residuals are the sum of squared ϵ as described in S.4.2. The network of the Orton Model contains 13 edges, which is marked in red.

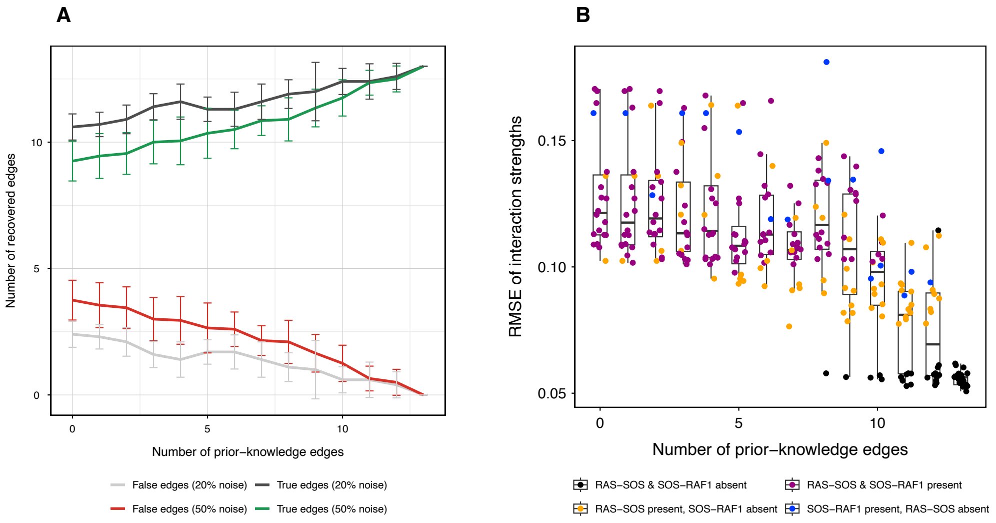

Figure S2: scMRA network reconstruction with prior network information. (**A**) Number of true and false edges in scMRA-reconstructed networks of the Orton model. We simulated 500 cells with 20% and 50% of noise. We randomly sampled true edges from the Orton model, used them as prior-knowledge in scMRA and recovered additional edges to reconstruct networks with 13 edges in total. The number of edges is an average across 5 simulations. Error bars represent standard deviations from the mean. Especially when the level of noise is high prior-knowledge information can guide scMRA and recover more true and less false edges. (**B**) RMSE of interaction strengths for models with an increasing number of prior-knowledge edges. We simulated 500 cells with 50% noise (the coloured lines in A) and repeated the simulation 20 times for every number of prior-knowledge edges. Prior-knowledge information does not only increase the number of true edges that we recover (A), but it also improves the strengths of the recovered interactions. Every dot represents a simulation and the colour depicts the presence of two frequently occurring false-positive edges (Fig. S1A). The presence of ether one of these two false-positive edges deteriorates the recovery of interaction strengths. E.g., Fig. 2C shows that a reconstruction with these two false-positive edges also underestimates the true interaction between P90RSK and SOS.

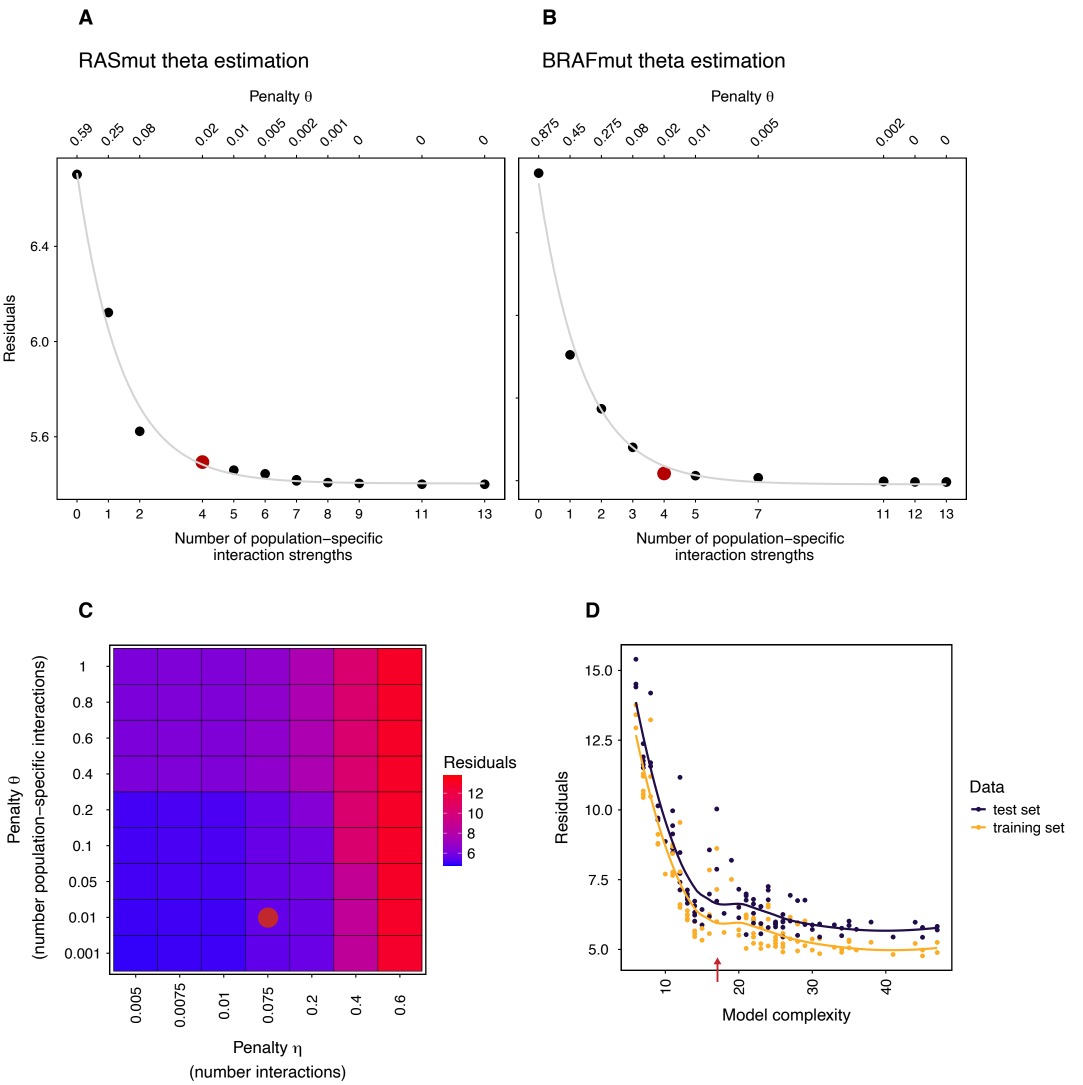

Figure S3: Model performance as a function of eta and theta. For the reconstruction of the RAS mutant network (**A**) and the BRAF mutant network (**B**) we chose the θ-parameter to reconstruct networks with 4 population-specific interaction strengths (red point). We first chose an η to reconstruct networks with 13 edges - the number of edges in the Orton model - and then varied the θ-parameter to find a simple network topology with low residuals. Residuals are the sum of squared ϵ as described in S.4.2. (**C**) A heatmap of the residuals of the scCNR-model for an array of η and θ-parameters 250 wildtype and 250 RAS-mutant cells and 20% noise. Presented values are an average of 5 simulations. The red point marks the average of simulations with network complexities (12 edges and 4 population-specific interaction strengths) that are closest to panel A and the reconstructions of the RAS mutant network that we used in the results (Fig. 3). (**D**) The residuals of panel C as a function of model complexity. In addition to the simulations in panel C (training set), we simulated a test set consisting of additional 250 wildtype and 250 RAS-mutant cells. We fixed the reconstructed network models obtained on the training set and evaluated the performance of these models on the test data set. Model complexity is the sum of indicator constraints (indicators for interactions and indicators for interactions that are population-specific). Every point represents a unique combination of η and θ-values (different combinations can have the same model complexity). The red arrow marks the chosen network complexity for our reconstructions (Fig. 3) (13 edges and 4 population-specific interaction strengths).

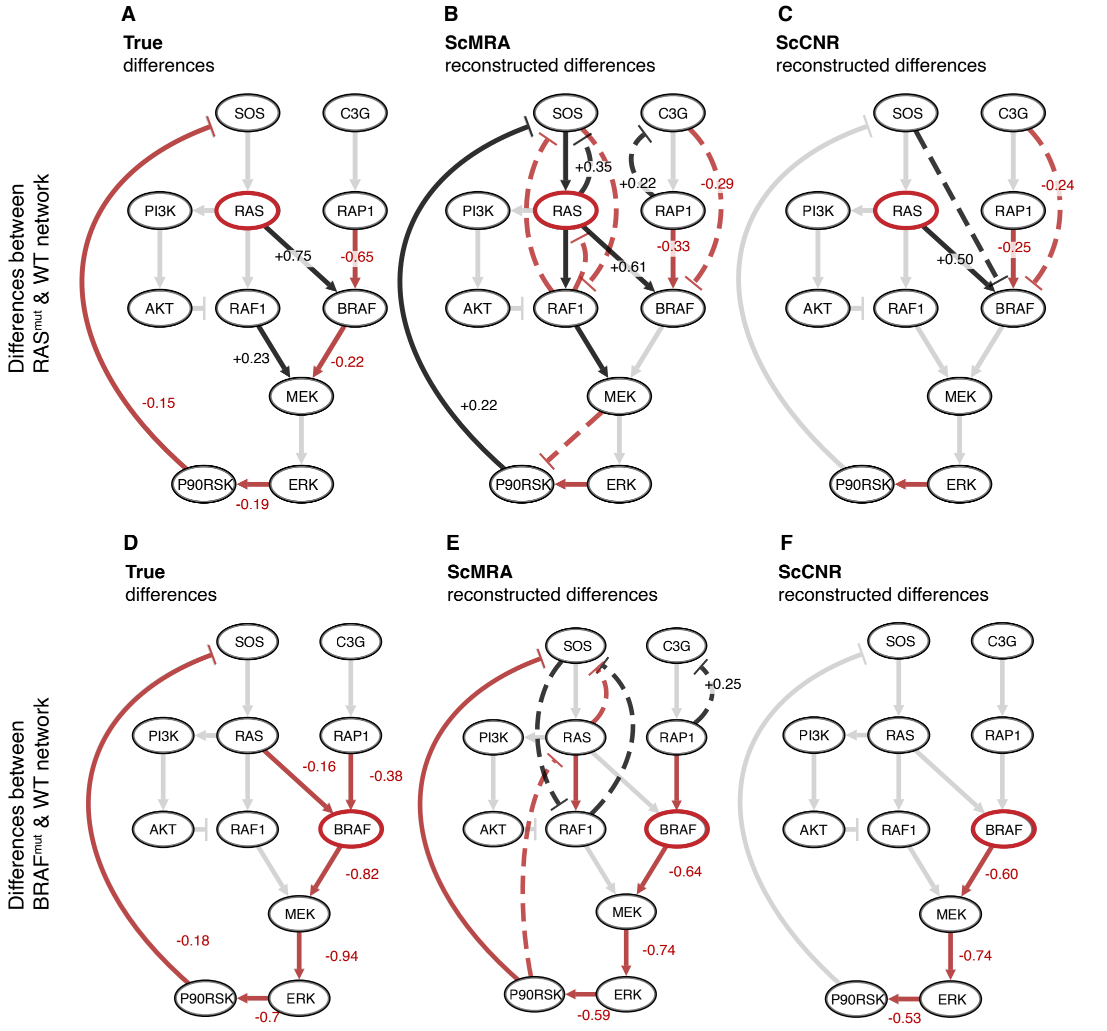

Figure S4: scCNR network reconstruction with high noise (50%). The first row shows network differences between the Ras mutant and wild-type population, the second row between the BRAF mutant and wild-type population. From left to right population-specific interactions in the network for the Orton model (**A,D**), combined scCNR reconstruction (**B,E**), and two separate scMRA reconstructions (**C,F**). Every network was reconstructed from 20 different simulations and interaction strengths were averaged. Interactions that are stronger in the mutant compared to the wild type are in black and edges with an decreased strength are in red. Edges with a strength-difference above 0.1 are visualized and differences above 0.2 are annotated. Dashed lines represent false positive edges. The true network topology is depicted in gray in the background.

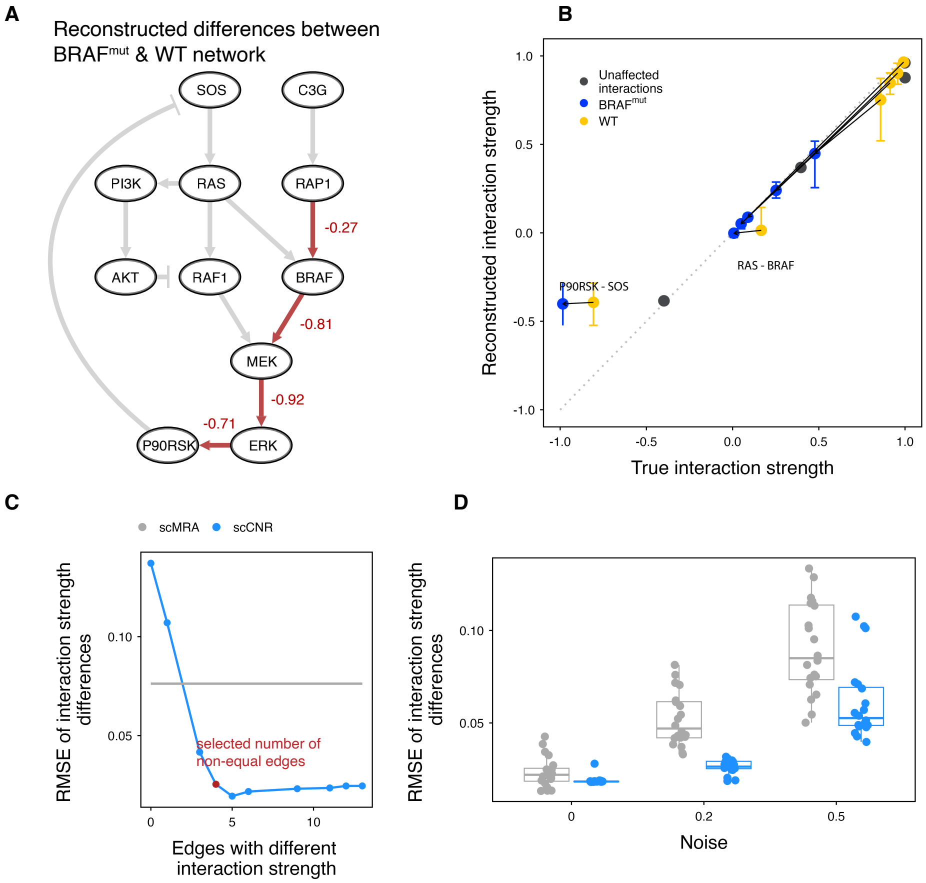

Figure S5: Reconstructing signaling differences between the BRAF mutant and wild-type cell population. (**A**) Reconstructed differences between the BRAF mutant and wild-type network from two exemplary cell populations of 250 cells with 20% noise. In grey in the background is the true network topology. In black/ red positive and negative differences of the BRAF mutant network compared to the wild-type network are highlighted. (**B**) Correlation of reconstructed (average of 20 simulations with 250 cells per population and 20% noise) versus true interaction strength for edges in the BRAF mutant (blue), wild-type (yellow) population and in grey edges with the same interaction strength in both populations (difference below 0.1). Error bars on every point visualize maximum variation of the reconstructed interaction strengths. Black lines connect the wild type and mutant interaction strengths. (**C & D**) Reconstruction of differences between wild type and mutant cell population measured by RMSE of interaction strength differences. (C) Effect of modelling increasing number of non-equal interaction strengths on the RMSE of interaction strenght differences. (D) RMSE of differences for joined/ separate reconstructions of the network with various levels of noise.

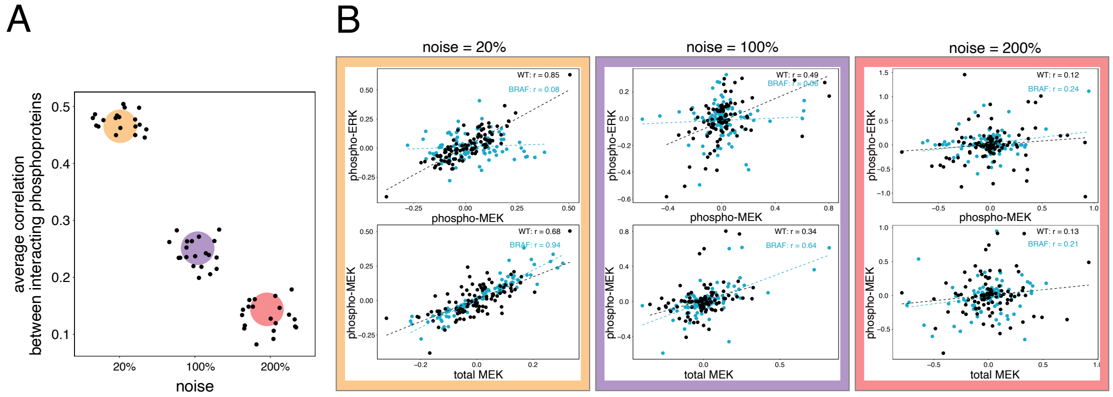

Figure S6: Simulations with increased noise reduce correlations between proteins. (**A**) The average correlation between interacting phospho-proteins for various noise levels added to the data. We simulated 100 cells of the wild-type and BRAF-mutant populations each, added noise (20%, 100% and 200%) on the simulated data, and repeated the simulations 20 times. (**B**) Example correlations between phospho-ERK and phospho-MEK (top panels), and between phospho-MEK and total MEK (bottom panels) under the three noise conditions (differerent colored boxes matching the circles in Panel A). Protein abundances of wild-type cells are colored in black and of the BRAF-mutant cells in turquoise.

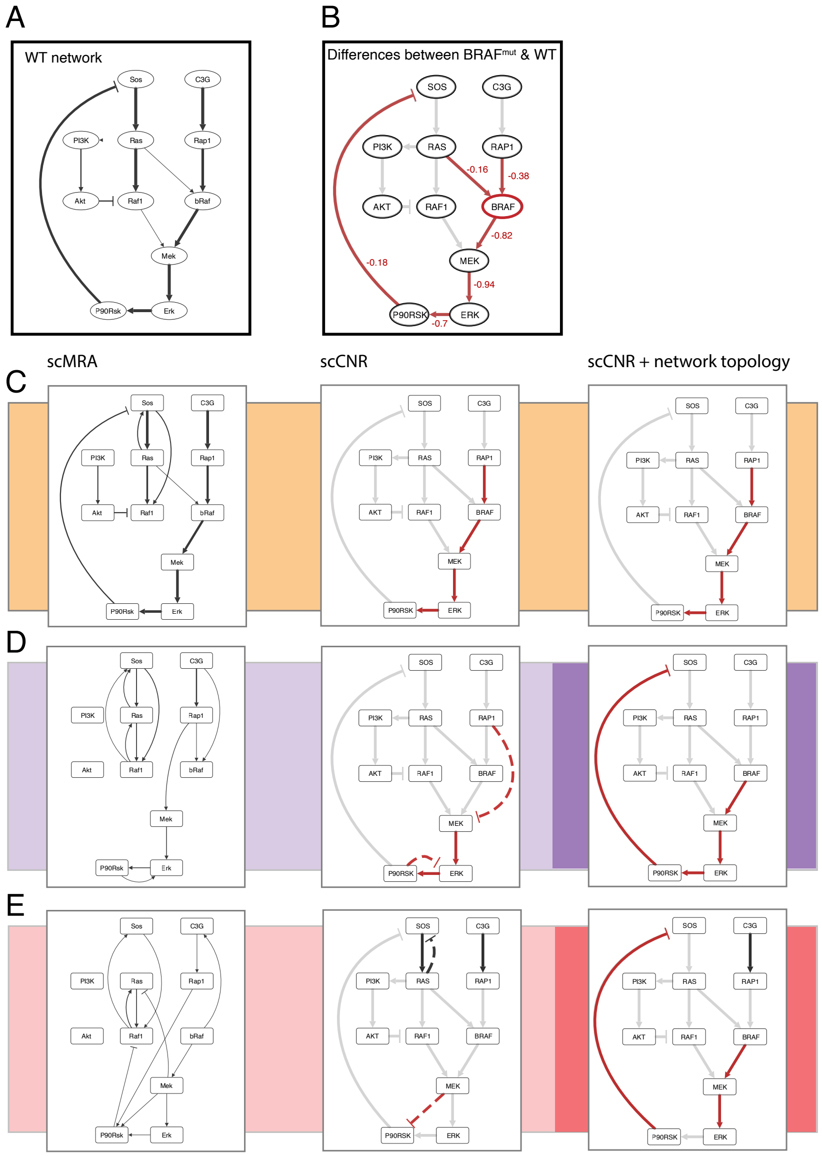

Figure S7: scCNR recovers population differences even under high noise conditions when guided by the network topology. (**A**) The true wild-type network topology from the Orton model. The width of the edges represents the interaction strength. (**B**) True differences between the wild-type and the BRAF-mutant network. Black edges have a higher interaction strength in the mutant, and red edges have a weaker interaction strength in the mutant. Gray edges in the background represent the true network topology as in A. (**C, D, E**) Network reconstructions for the three noise levels from Fig. S6 (C: noise = 20%, D: noise = 100%, E: noise = 200%). Reconstructions are based on data from 20 simulations of either the wild-type network with scMRA (*de* novo reconstruction), the network and its differences between the two populations with scCNR (*de* novo reconstruction), or the differences between populations with scCNR guided by the network topology of the Orton model. The depicted networks are an average of the 20 reconstructions. For scMRA we show the strongest 13 edges across all reconstructions and for scCNR we show the 4 strongest differences across all reconstructions. The scMRA networks show the recovered interaction strengths of the wild-type cells only and the networks for scCNR show the the interaction strength differences of the BRAF-mutant network compared to the wild-type network as in A and B. The decreased opacity of the colored background shows where scMRA/scCNR fails to recover the majority of interactions/interaction strength differences. Under low noise conditions scMRA recovers the topology well and scCNR recovers true differences. Under high noise conditions, where scMRA fails to recover the network topology *de* novo, scCNR can still recover meaningful network differences when guided by the network topology.

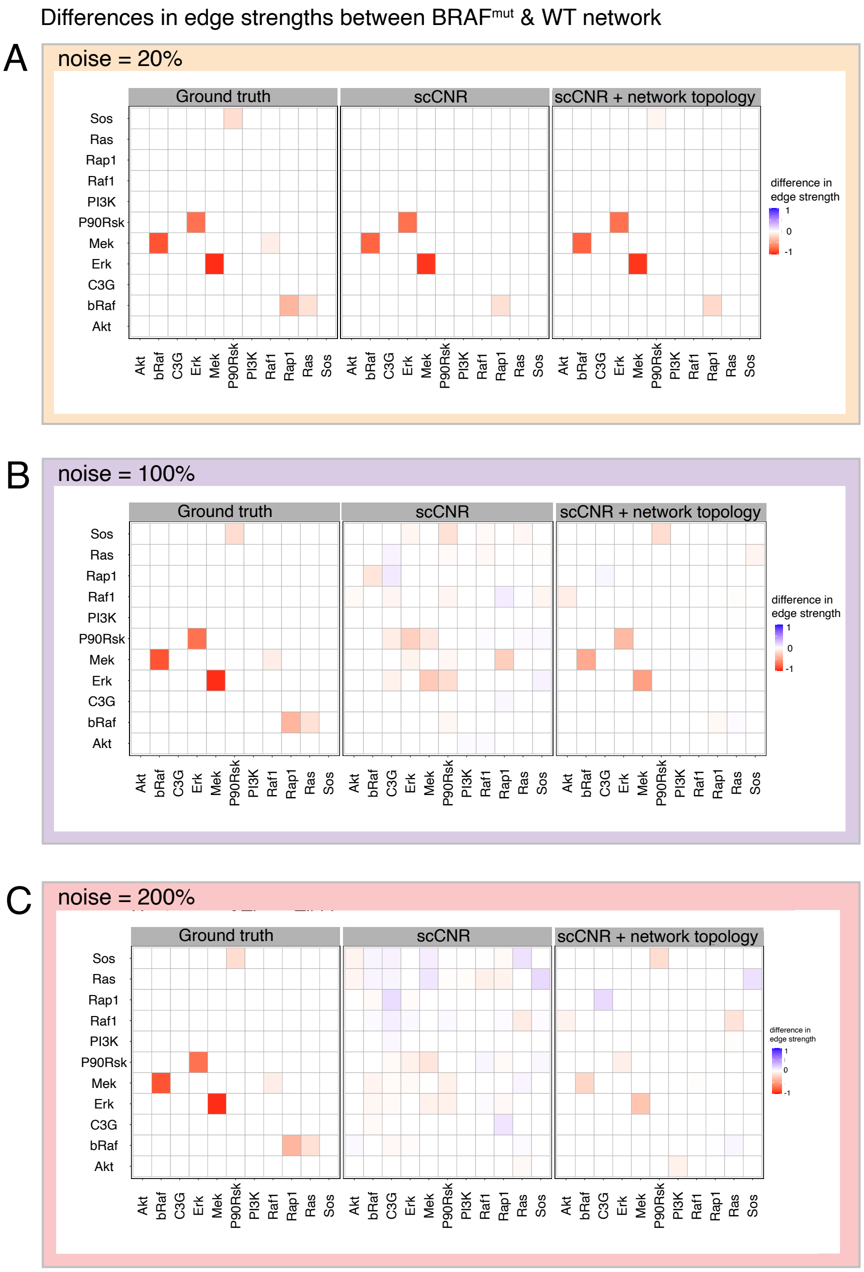

Figure S8: Heatmaps of the interaction strength differences between the BRAF-mutant and wild-type networks recovered with scCNR. The interaction strength differences for the networks in Fig. S7 with (**A**) noise = 20%, (**B**) noise = 100% and (**C**) noise = 200%. Depicted are the ground truth (left), interaction strength differences for the networks recovered with scCNR without prior network information (center) and the differences for scCNR guided by the network topology (right). All heatmaps are an average of the 20 simulations in Fig. S7. Differences between populations can not be recovered by scCNR with a noise level of 100% (B). When guided by the network topology scCNR can still recover meaningful differences even with noise of 200%.

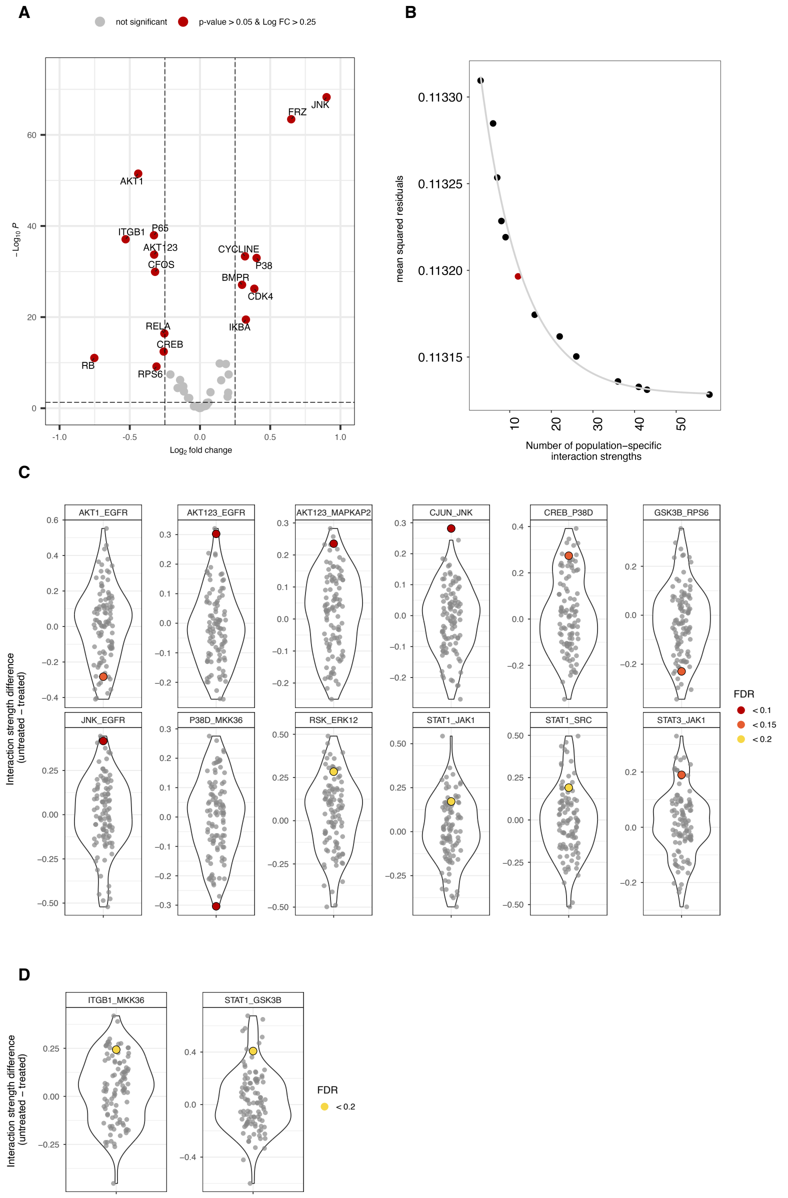

Figure S9: Analysis of signaling differences between untreated and EGFR-inhibitor treated keratinocytes. (**A**) Volcano plot of (phospho)-proteins from the ID-seq panel. Proteins with a corrected p-value below 0.05 and $\log_2$ fold change higher than 0.3 are annotated. (**B**) Mean squared residuals of the scCNR analysis for an increasing number of population-specific interaction strengths. (**C**) Permutation analysis to estimate significance of detected edge differences in our intial scCNR analysis. We randomly permuted cell population labels (treated/untreated), fixed edges that were found to be different in the original data and repeated scCNR 100 times. Color coded dots represent the originally recovered interaction strength differences and their false discovery rate. (**D**) Permutation analysis of the two edges that were additionally identified to be different in the scMRA-extended network.

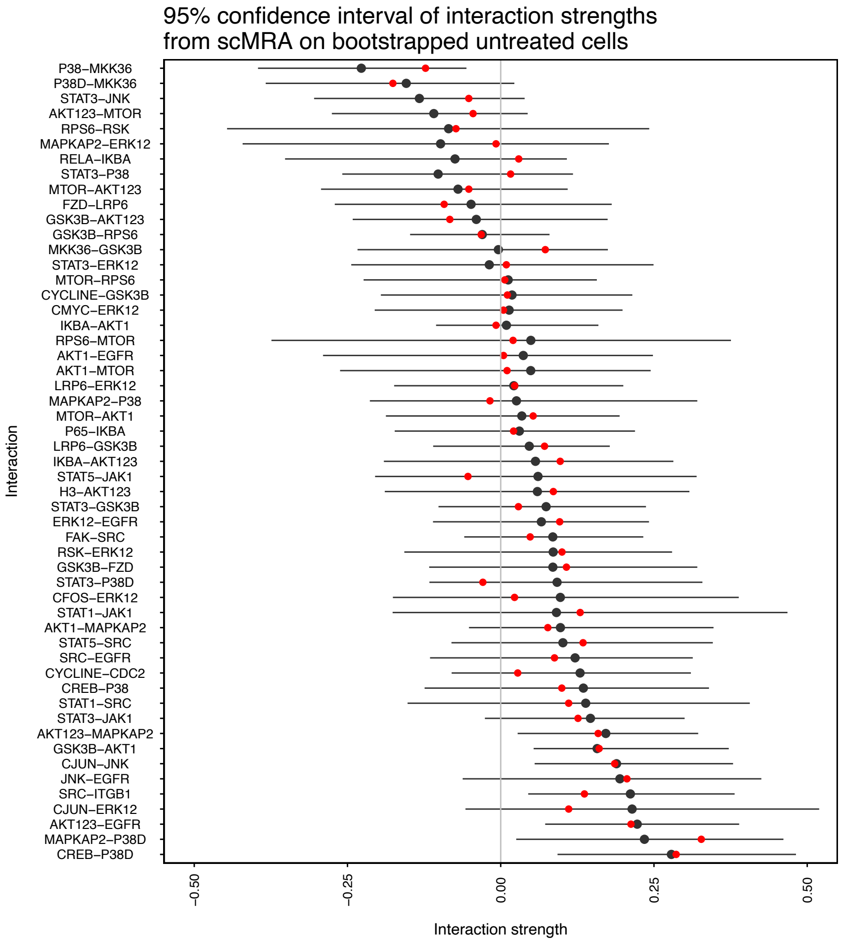

Figure S10: Interaction strength variation of prior knowledge edges in the control population. We conducted 100 bootstrap analyses on the control cells of scID-seq dataset and applied scMRA using the prior knowledge topology as illustrated in (Fig. 4C) to get interaction strength distributions for each edges. The horizontal lines indicate the 95% confidence interval for each interaction strength. The red dots indicate the edge strength obtained for the control population when applying scCNR on the entire dataset.

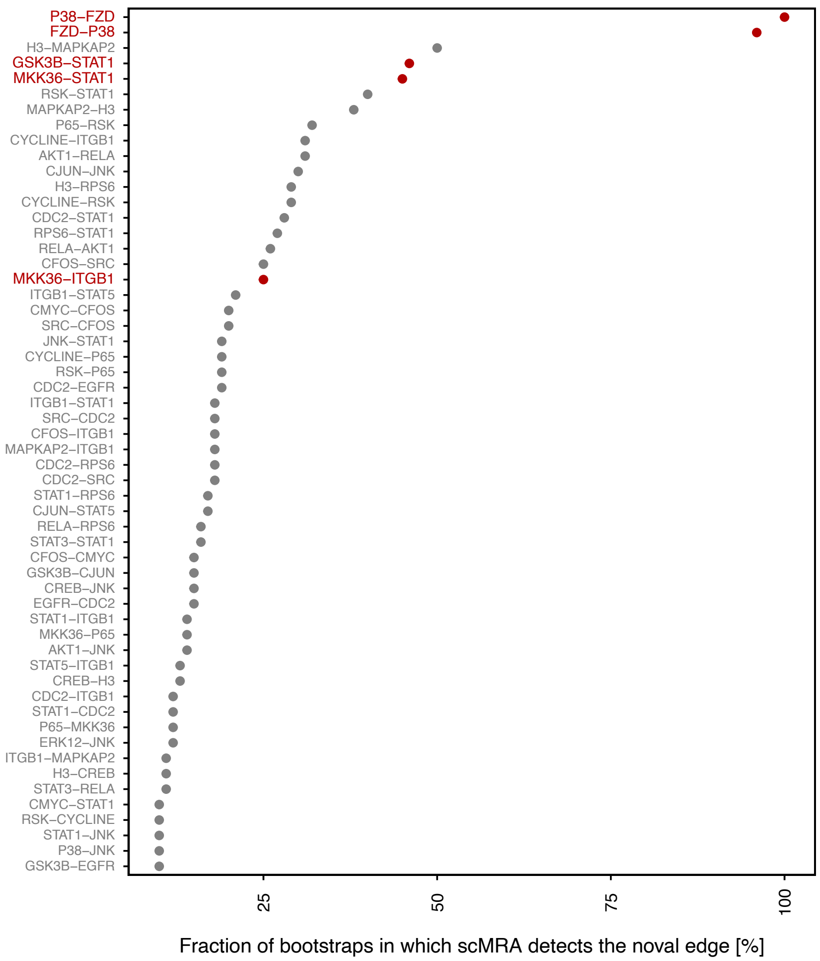

Figure S11: Presence of novel edges added by scMRA in bootstraps. Fraction of bootstraps (out of 100) in which an edge is added to the prior-network topology when applying scMRA to the untreated cells of the scID-seq dataset. We used the same value for η as in Fig. 4 to recover new edges in the network. Only edges that occur in 10 or more bootstraps are included in the figure. Edges that were added in the initial application of scMRA are highlighted in red.
